# Psychological and social factors influencing AI painting tool adoption among young-old adults in the context of creative aging

**DOI:** 10.3389/fpsyg.2026.1852192

**Published:** 2026-05-26

**Authors:** Meichen Fang, Wei Yang, Yanjun Tan, Junping Xu, Jun Yin

**Affiliations:** 1 School of Digital Technology and Innovation Design, Jiangnan University, Wuxi, China; 2 School of Design, Guangxi Arts University, Nanning, China; 3 College of Media and International Culture, Zhejiang University, Hangzhou, China; 4 Future Imaging Laboratory, Innovation Center of Yangtze River Delta, Zhejiang University, Jiaxing, China

**Keywords:** AI painting tools (AIPT), creative aging, digital inclusion, intergenerational support, technology acceptance model (TAM)

## Abstract

**Introduction:**

AI painting tools (AIPT) are creating new opportunities for creative aging, digital inclusion, and social participation among young-old adults. However, the psychological and social factors associated with their adoption remain underexplored. This study examined the factors associated with intention to use AIPT among adults aged 60–69 by extending the Technology Acceptance Model (TAM) with technological characteristics, social support, and individual motivational factors.

**Methods:**

Data from 450 valid respondents were analyzed using partial least squares structural equation modeling.

**Results:**

The results showed that output quality (OQ), relative advantage (RA), and trialability (TR) were positively associated with perceived usefulness (PU), with OQ showing the strongest association. Perceived ease of use (PEOU) was associated with RA, intergenerational support (IS), peer influence (PI), hedonic motivation (HM), and personal innovativeness (PIN), highlighting the importance of technological simplification, social support, and motivational readiness in older adults’ digital creative engagement. PEOU also showed a stronger association with intention to use (IU) than PU, suggesting that usability may be particularly salient in AIPT acceptance among young-old adults.

**Discussion:**

By situating technology acceptance in the context of creative aging, this study advances understanding of age-specific digital adoption and emphasizes the practical importance of age-friendly design, high-quality AI-generated output, and IS in supporting later-life creativity and inclusion.

## 1 Introduction

According to China’s national population statistics at the end of 2024, the population aged 65 and above had exceeded 220 million, accounting for 15.6% of the total population, indicating that China is gradually entering a moderately aged society (Xiang and Xing, 2025). Within this broader aging context, adults aged 60–69 constitute an important early-stage older adult group. In the context of China’s social and retirement system, age 60 commonly marks an important transition into later life. Therefore, this study operationally defines “young-old adults” as individuals aged 60–69 years. Compared with the oldest-old population, this group generally has relatively better physical conditions and tends to demonstrate a stronger willingness to engage in social activities and daily participation (Liu et al., 2022). However, life-course transitions such as retirement, the independence of adult children, and the death of a spouse may increase their risk of loneliness, depression, and other negative psychological states, as well as broader mental health stress (Reynolds, 2010). Therefore, how to provide this group with accessible, convenient, meaningful, and participatory activities has become an important issue in current aging research.

Creative engagement is an important form of active aging and meaningful later-life participation. Prior studies suggest that art-based activities may support older adults’ self-expression, emotional well-being, self-efficacy, social connection, and continued creative development (Cho and Chang, 2024; Du et al., 2023; Fisher and Specht, 1999). However, traditional offline art activities, such as painting, crafts, calligraphy, and art therapy, often depend on physical access, artistic skills, professional facilitation, and context-specific adaptation, which may limit their accessibility and scalability for older adults with different physical conditions, cognitive capacities, cultural preferences, and personal interests (Evans et al., 2022; Löfgren, 2021). Therefore, more accessible and flexible forms of creative engagement are needed.

The rapid advancement of artificial intelligence (AI) has created new possibilities for accessible creative engagement, particularly through AI-assisted creative tools. Compared with traditional art activities and conventional digital drawing software, AIPT can lower the operational threshold of visual creation by allowing users to generate images through simplified inputs, such as text prompts or sketches (Xu et al., 2023), providing personalized feedback tailored to individual abilities, and facilitate flexible engagement for older adults regardless of time and location (Patil, 2024). Recent studies have demonstrated that AI tools can support older adults in adapting to age-related changes while enhancing their sense of autonomy and social connection. In elderly care settings, these tools have been observed to nurture imagination, strengthen interpersonal bonds, and improve overall psychological well-being, thus serving as an effective carrier for enriching the spiritual life of the elderly and promoting digital inclusion (Iqbal et al., 2025).

Despite the growing relevance of AIPT for creative aging and digital inclusion, existing research remains fragmented across three related but insufficiently connected streams. First, creative aging research has established that artistic and creative engagement can support older adults’ psychological well-being, self-expression, and social participation. However, this literature has mainly examined traditional or offline forms of creative activity, such as painting, crafts, calligraphy, and art therapy, while paying limited attention to how AI-driven creative tools may reshape creative participation in later life (Han and Yang, 2018). Second, research on the acceptance of AI in painting primarily targets younger professionals (such as designers, art students, and art teachers), neglecting the developmental potential of older adults in this field and their unique cognitive characteristics and needs (Lee and Lee, 2019; Shen et al., 2024). These studies have provided valuable insights into the usability, productivity, and creative potential of AI-assisted tools, but they have not sufficiently examined whether and how older adults perceive such tools as accessible, meaningful, and acceptable for everyday creative engagement. Third, research on older adults’ technology acceptance has widely applied TAM (Harris and Rogers, 2023) and its extensions to digital devices, mobile applications, companion robots, and other functional technologies. Nevertheless, existing research pays limited attention to older adults’ adoption of creative AI. Hence, this study integrates technological, social and personal factors to unpack the acceptance mechanisms of such technologies.

To fill these gaps, this study focuses on young-old adults (the core potential users of AIPT) and constructs an integrated theoretical model by integrating the TAM with factors from three dimensions: technological characteristics, social support, and individual motivation. Based on 450 valid questionnaire samples, this study employs Partial Least Squares-Structural Equation Modeling (PLS-SEM) to address three core research questions:

1. What factors are associated with young-old adults’ intention to use AIPT?
2. Through what internal acceptance pathways are the various factors associated with young-old adults’ intention to use AIPT?
3. What practical implications do the identified acceptance pathways have for AI developers and social organizations in supporting age-friendly AIPT design and digital inclusion among older adults?

This study makes three main contributions. First, it extends TAM-based research to the underexamined context of AIPT adoption among young-old adults, thereby shifting the focus of older adults’ technology acceptance research from primarily functional digital technologies to AI-assisted creative tools. Second, it enriches the creative aging literature by moving beyond traditional offline art participation and examining how AI-assisted visual creation may support later-life creative engagement, self-expression, and digital inclusion. Third, by integrating technological characteristics, social support, and individual motivational factors, this study identifies distinct belief-formation pathways through which young-old adults evaluate AIPT, offering practical implications for age-friendly interface design, onboarding support, and socially supported learning.

## 2 Theoretical background and hypotheses

### 2.1 Creative engagement and AIPTs in later life

Creative engagement plays a critical role in supporting psychological well-being and social participation in later life (Edwards and Owen-Booth, 2021; Ma et al., 2023). Through activities such as drawing, music, and crafts, older adults can maintain cognitive stimulation, express personal experiences, and reinforce a sense of identity and autonomy (Cho and Chang, 2024). Prior studies have consistently shown that participation in creative activities is associated with reduced loneliness, improved emotional well-being, and enhanced life satisfaction (Bell et al., 2013). Therefore, enabling accessible forms of creative participation is considered an important pathway for promoting active and healthy aging. However, traditional art activities often involve substantial barriers, including the need for prolonged skill acquisition, physical dexterity, and access to specialized materials (Hajek et al., 2025). These constraints may limit participation, particularly among older adults without formal artistic training. As a result, the accessibility of creative engagement largely depends on whether tools can reduce these barriers while maintaining meaningful output (Hennelly et al., 2019).

AIPT introduce a new form of creative participation by enabling users to generate visual content through simplified interactions such as text prompts and sketch-based inputs (Bomba and De Angeli, 2025). Compared with conventional digital drawing tools, AIPT shift the creative process from manual skill execution to concept-driven interaction (Linden et al., 2025). This transformation has two important implications for older adults’ technology evaluation. On one hand, AIPT may enhance the perceived advantages of creative tools by reducing effort requirements and enabling rapid generation of visually meaningful outputs (Zhang et al., 2025). Features such as immediate visual feedback and flexible trial processes allow users to experiment with different ideas at low cost (Shojaei et al., 2024). These characteristics are likely to associate with users’ perceptions of relative advantage, trialability, and output quality, which are critical dimensions in evaluating emerging technologies (Russo, 2024). On the other hand, AIPT also introduce new interaction challenges. The use of prompt-based generation, unfamiliar interface structures, and uncertainty in AI-generated outcomes may increase cognitive load and perceived complexity, particularly for users with limited digital experience (Tankelevitch et al., 2024). In this context, ease of use becomes a central concern influencing whether users feel capable of engaging with the system.

Taken together, the adoption of AIPT among older adults is not determined solely by the availability of creative opportunities, but by how users evaluate the technology across multiple dimensions, including its perceived benefits, ease of interaction, and experiential qualities (Jeong et al., 2025). In addition, given the social and motivational characteristics of older adults, external support and individual differences may further shape these evaluations (Zhengkun et al., 2025). Therefore, creative aging and digital inclusion are used in this study to define the research context and practical relevance of AIPT adoption, while the explanatory model itself is grounded in an extended TAM framework.

### 2.2 Technology acceptance model (TAM)

The TAM, originally proposed by Davis (1989), explains individuals’ engagement with information systems through two central cognitive beliefs: perceived usefulness (PU) and perceived ease of use (PEOU). PU refers to the extent to which a system is perceived as enhancing performance, whereas PEOU reflects the degree to which system interaction is perceived as effortless. These constructs jointly are associated with users’ attitudes and behavioral intentions toward technology use (Malatji et al., 2020). Extensive empirical research across information systems contexts has confirmed the stability of the relationships among PU, PEOU, and behavioral intention, while also extending the model with contextual variables to better capture domain-specific influences (Huang and Yang, 2025).

Key extended versions of TAM include TAM2 (Venkatesh and Davis, 2000), TAM3 (Hamadeh et al., 2025), and UTAUT (Li, 2025), which provide detailed insights into emerging technology acceptance. Existing studies have fully demonstrated TAM’s flexibility and adaptability by extending or integrating it with other theories to explore user acceptance of various emerging technologies, covering virtual reality learning, mobile applications, the metaverse, generative AI tools (e.g., ChatGPT, GenAI-Image), and other fields (Ali et al., 2025; Damilig, 2025). These studies consistently confirm that TAM and its extended versions retain strong predictive power across different technological contexts, and appropriate integration with other theoretical frameworks can further improve the explanatory power of the model for specific technology acceptance behaviors (Li et al., 2025).

In recent years, TAM has been increasingly applied to elderly technology acceptance research, with relevant studies (Zin et al., 2023) consistently taking TAM and its extended models as the core analytical framework to explore elderly users’ acceptance of diverse digital technologies, including smartphones, virtual reality sports games, mobile music platforms, and companion robots (Liu M. et al., 2025). Some of these studies further optimize the model by combining other theories, while all focus on the core role of TAM’s key constructs (PU, PEOU) in explaining elderly technology acceptance, verifying TAM’s good applicability to the elderly user group (Yang et al., 2023). Given TAM’s strong predictive power in explaining emerging technology acceptance and its emphasis on system usability, the model is well suited to explaining older adults’ acceptance of AIPT because PU and PEOU capture the two core concerns of practicality and usability that are particularly salient in older adults’ adoption of digital technologies (Martín-García et al., 2021).

### 2.3 Proposed model and hypotheses

#### 2.3.1 Technological characteristics

##### 2.3.1.1 Relative advantage (RA) and trialability (TR)

RA and TR originate from the Diffusion of Innovation (DOI) theory proposed by Everett M. Rogers, which explains how innovations are adopted within social systems. RA refers to the degree to which a technology is perceived as superior to existing alternatives (Rondan-Cataluña et al., 2015). In the context of AIPT, RA reflects older adults’ perception that AIPT enables more convenient, flexible, and enjoyable artistic expression compared with traditional art practices constrained by time, location, or technological skill requirements (Binyamin and Zafar, 2021).

TR describes the extent to which a technology can be experimented with prior to full adoption (Tuomi et al., 2023). For older adults, opportunities to explore AIPT in a low-risk environment reduce uncertainty and technology-related anxiety (Talantis et al., 2020). Experiential trial processes allow users to evaluate whether AIPT aligns with their personal interests and capabilities, thereby facilitating technology acceptance. Based on these arguments, the following hypotheses are proposed:

*H1a*: RA is positively associated with PU of AIPT among young-old adults.

*H1b:* RA is positively associated with PEOU of AIPT among young-old adults.

*H2a:* TR is positively associated with PU of AIPT among young-old adults.

*H2b:* TR is positively associated with PEOU of AIPT among young-old adults.

##### 2.3.1.2 Output quality (OQ)

OQ refers to users’ evaluation of the generated outputs themselves, particularly in terms of their visual quality, coherence, aesthetic appeal, and perceived keep/share value, as discussed in the Information Systems Success Model (ISSM) (DeLone and McLean, 2003). In intelligent systems, OQ represents a critical determinant influencing users’ cognitive evaluation and continued system usage. In generative artificial intelligence environments, system outputs are directly translated into visual creative results. Unlike traditional digital tools that require advanced operational skills, AIPT provides immediate visual feedback through automated content generation (Wang, 2008). For older adults with limited artistic training, the perceived quality of generated artworks is closely associated with their confidence, satisfaction, and willingness to engage in creative activities (Alzahrani et al., 2019). High-quality AI-generated images—characterized by visual coherence, aesthetic appeal, and semantic relevance to user prompts—may strengthen users’ confidence that the system can generate visually satisfactory and intention-consistent outcomes (Yoon and Kim, 2023). When users perceive that AI outputs successfully transform their ideas into meaningful visual outcomes, they may be more likely to view the system as supportive of their creative goals and to experience the interaction process as more manageable (Kaimal et al., 2019). In the present study, OQ refers specifically to the perceived merit of the generated outputs themselves, rather than to the enjoyment experienced during interaction or the instrumental utility of the system. From a sustainable digital participation perspective, higher OQ may reduce frustration during interaction and lower barriers to continued engagement, thereby supporting inclusive creative participation among older adults. Accordingly, the following hypotheses are proposed:

*H3a*: OQ is positively associated with PU of AIPT among young-old adults.

*H3b:* OQ is positively associated with PEOU of AIPT among young-old adults.

#### 2.3.2 Social support

##### 2.3.2.1 Intergenerational support (IS)

IS plays an important role in facilitating older adults’ adoption of emerging technologies within family contexts (Jin and Qu, 2025). Assistance provided by younger family members, including operational guidance and technical explanation, functions as a social-cognitive resource that reduces perceived complexity and enhances technology-related self-efficacy (Jokisch et al., 2022). According to Social Cognitive Theory (SCT), such support can improve users’ understanding of system operation and strengthen confidence in technology use, thereby lowering perceived difficulty (Serrano, 2018). Moreover, explanatory support regarding functional benefits and usage value helps older adults better recognize the utility of new technologies (Liu et al., 2022; Zhu et al., 2023). Therefore, IS is expected to be positively associate with older adults’ perceptions of both PU and PEOU of AIPT. Accordingly, the following hypotheses are proposed:

*H4a:* IS is positively associated with PU of AIPT among young-old adults.

*H4b:* IS is positively associated with PEOU of AIPT among young-old adults.

##### 2.3.2.2 Peer influence (PI)

PI refers to the impact of individuals within the same social group on users’ perceptions and technology-related behaviors. For older adults, peer experiences and recommendations serve as an important source of social learning when evaluating unfamiliar digital technologies (Mei, 2024). Prior studies have shown that observing peers’ successful technology use enhances individuals’ recognition of technology value and reduces uncertainty toward adoption. In the context of AIPT, positive peer participation and shared creative experiences may strengthen older adults’ perceptions of the benefits derived from AIPT (Collazo-Castiñeira et al., 2025). Therefore, PI is expected to enhance PU. In addition, peer interaction facilitates experiential learning and informal assistance, which can reduce perceived operational difficulty and technology-related anxiety among older users (Bhowmick, 2023). Evidence suggests that peer-supported learning environments improve confidence in using digital systems and increase PEOU. Accordingly, PI is expected to be positively associated with PEOU of AIPT. Accordingly, the following hypotheses are proposed:

*H5a:* PI is positively associated with PU of AIPT among young-old adults.

*H5b:* PI is positively associated with PEOU of AIPT among young-old adults.

#### 2.3.3 Individual motivation

##### 2.3.3.1 Hedonistic motivation (HM)

HM refers to the intrinsic enjoyment, pleasure, and emotional gratification that motivate individuals to engage with a technology (Ramírez-Correa et al., 2018). As an individual motivational factor, HM reflects users’ internal desire to participate in a technology-mediated activity because the experience itself is enjoyable and satisfying. Prior technology acceptance studies have shown that enjoyment is an important intrinsic driver of users’ cognitive evaluations and adoption behaviors, particularly in emotionally engaging and creative technology contexts (Lu et al., 2025). In the context of AIPT, older adults may experience enjoyment not only because the technology supports creative expression, but also because it enables playful exploration, low-pressure experimentation, and a sense of personal engagement in the creative process (Syed-Abdul et al., 2019). When users find the creative interaction enjoyable, they are more likely to form positive evaluations of the technology and to perceive it as beneficial for their goals, thereby strengthening PU (Lai et al., 2025). In addition, enjoyable experiences may reduce psychological resistance and perceived cognitive burden, making the interaction process feel less difficult and thereby enhancing PEOU (Wang et al., 2025). Studies on AI and digital technology adoption further suggest that pleasurable interaction can improve user confidence and facilitate PEOU, especially among older adults who may experience technology-related anxiety (Hao and Cui, 2025). Therefore, HM is expected to be positively associated with PEOU of AIPT. Accordingly, the following hypotheses are proposed:

*H6a:* HM is positively associated with PU of AIPT among young-old adults.

*H6b:* HM is positively associated with PEOU of AIPT among young-old adults.

##### 2.3.3.2 Personal innovativeness (PIN)

PIN is commonly defined as an individual’s willingness to experiment with new information technologies and explore novel technological applications independently. Importantly, prior research emphasizes that innovativeness is domain-specific, meaning that individuals may demonstrate different levels of innovativeness depending on the technological or creative context involved (Agarwal and Prasad, 1998). In digital creative environments, PIN reflects users’ readiness to explore AIPT, experiment with visual generation processes, and engage in novel forms of technology-mediated artistic expression (Hu et al., 2025). Individuals with higher innovativeness toward digital art creation are therefore more likely to recognize the creative benefits of AI tools and perceive them as valuable for supporting artistic exploration and self-expression (Zhao and Fu, 2024). Moreover, domain-related innovativeness facilitates exploratory learning behaviors and reduces psychological barriers when interacting with unfamiliar creative technologies (Ma et al., 2025). Older adults who possess stronger innovative tendencies toward digital artistic activities are more willing to engage in experimentation and iterative interaction (Lai et al., 2025), thereby lowering perceived operational difficulty and enhancing usability perception during AI-assisted creation processes. Accordingly, the following hypotheses are proposed:

*H7a:* PIN is positively associated with PU of AIPT among young-old adults.

*H7b:* PIN is positively associated with PEOU of AIPT among young-old adults.

#### 2.3.4 Core TAM variables

##### 2.3.4.1 Perceived usefulness (PU) and perceived ease of use (PEOU)

PU refers to the extent to which individuals believe that using a technology enhances task performance, whereas PEOU represents the degree to which a technology is perceived as effortless to learn and operate. According to the TAM (Siette et al., 2024), PU and PEOU are fundamental determinants influencing users’ behavioral intentions toward technology adoption. Previous studies in AI-related contexts have consistently confirmed that systems perceived as useful and easy to use significantly increase users’ acceptance intentions (Xia, 2025).

For older adults, these factors become particularly critical due to age-related declines in digital learning confidence and operational adaptability (Rispler et al., 2025). In the context of AIPT, these constructs are closely related to the creative process enabled by generative technologies. Unlike traditional digital drawing software that requires advanced artistic skills, AIPT allow users to generate visual artworks through simple textual prompts, sketch inputs and iterative interaction with the system (Wijesundara and Xixiang, 2018). When older adults perceive that such tools can effectively support creative expression, help them visualize ideas, or support idea visualization, creative goal attainment, and more efficient task completion, their PU of the system increases (Jeong and Yoo, 2007).

At the same time, ease of interaction plays a critical role in older adults’ engagement with AIPT. These systems often involve novel interaction mechanisms such as prompt-based generation, parameter adjustment, and iterative image refinement. If these interaction processes are perceived as intuitive and easy to understand, older users may experience lower cognitive barriers and greater confidence in exploring creative possibilities. Consequently, higher PEOU can enhance PU and directly strengthen older adults’ willingness to adopt AIPT.

##### 2.3.4.2 Intention to use (IU)

Behavioral intention is commonly regarded as the most immediate predictor of actual technology usage in technology acceptance research. In the context of AIPT-based digital art creation, IU reflects older adults’ willingness to adopt and engage with AIPT as part of their daily activities (Hwang et al., 2025).

For older adults, the motivation to use such tools is closely related to the opportunities for creative participation and meaningful self-expression that AIPT provide (Adams-Price and Morse, 2025). When older adults perceive that AIPT can help them express their thoughts, create meaningful visual works, or enrich their daily lives, they are more likely to be willing to use them (Pang and Cheng, 2023). Therefore, intention to use serves as a key outcome variable reflecting older adults’ acceptance of AIPT.

Accordingly, the following hypotheses are proposed:

*H8:* PEOU is positively associated with PU of AIPT among young-old adults.

*H9:* PU is positively associated with IU of AIPT among young-old adults.

*H10:* PEOU is positively associated with IU of AIPT among young-old adults.

**Figure 1:**
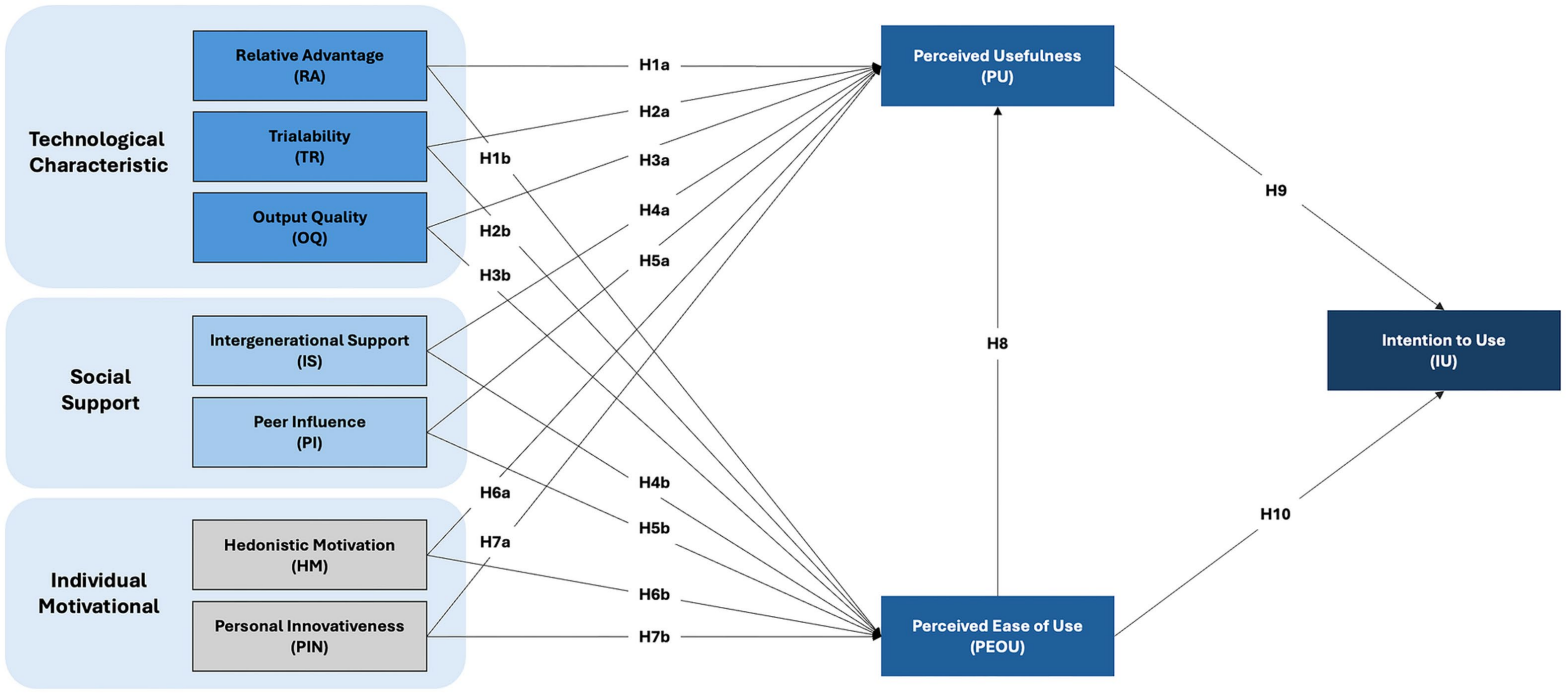
Proposed conceptual model.

The proposed model is shown in Figure 1.

The proposed model is structured as a TAM-centered extension rather than a broad multi-theoretical integration. TAM provides the core explanatory framework, in which PU, PEOU, and IU are treated as the main acceptance-related constructs. Other theories are used only as supporting lenses for selecting and explaining external variables. Specifically, DOI theory informs the inclusion of RA and TR; the ISSM supports the inclusion of OQ; and SCT helps explain the roles of IS and PI. In this way, technological characteristics, social support, and individual motivation are introduced as external antecedents of PU and PEOU in the specific context of AIPT adoption among young-old adults.

## 3 Methods

### 3.1 Participants

This study was conducted between July and November 2025, compliant with relevant guidelines and regulations. The target population of this study comprised young-old adults with basic internet proficiency. Inclusion criteria were established as follows: (1) aged 60–69 years; (2) able to understand Chinese and express personal perceptions clearly; (3) with prior experience using digital devices (e.g., smartphones, tablets, or computers) and a weekly usage frequency of ≥3 times; (4) voluntary participation in the study, provision of written informed consent, and willingness to complete the AI tool experience and subsequent questionnaire. Exclusion criteria were defined as follows: (1) severe cognitive impairment or related disorders; (2) any terminal illness.

From an ethical standpoint, this study was reviewed and approved by the Ethics Review Committee of the College of Media and International Culture, Zhejiang University (IRB No. cmic20260438). Informed consent was obtained from all participants prior to their participation. Participants were informed that their participation was voluntary, that they could withdraw from the study at any time, that their responses would be kept anonymous and confidential, and that all collected data would be used solely for academic research purposes with no commercial intent. No sensitive personal information, identifiable personal data, biological samples, medical records, or health-related intervention data were collected.

A total of 512 questionnaires were distributed in this survey, 483 were returned, and 450 valid samples were obtained after excluding 33 invalid questionnaires. Table 1 presents the demographic characteristics of the participants. Based on self-reported data, the sample shows a relatively balanced gender distribution, with 47.78% male and 52.22% female participants. In terms of age, most participants were aged 60–64 (61.78%), while 38.22% were aged 65–69. Regarding educational background, the largest group held undergraduate, college, or vocational college degrees (44.67%), followed by those with a high school education (35.56%), whereas 19.77% had a junior high school education or below. With respect to digital device usage experience, most participants reported regular use of digital devices for daily use (≥3 h/day) (42.00%), regular use (1–3 h/day) (26.22%), while fewer participants reported occasional use (0.5–1 h/day) (18.00%) or rare use (<0.5 h/day) (13.78%).

**Table 1:** Demographics of the participants (*n* = 450).

Measure	Items	Frequency	Percentage (%)
Age	60–64	278	61.78
	65–69	172	38.22
Gender	Male	215	47.78
	Female	235	52.22
Educational background	Junior high school and below	89	19.77
	High school	160	35.56
	Undergraduate/college/vocational college	201	44.67
Digital device usage experience	Daily use (≥3 h/day)	189	42.00
	Regular use (1–3 h/day)	118	26.22
	Occasional use (0.5–1 h/day)	81	18.00
	Rare use (<0.5 h/day)	62	13.78

### 3.2 Experimental platform and questionnaires administered

#### 3.2.1 Experimental platform

**Figure 2:**
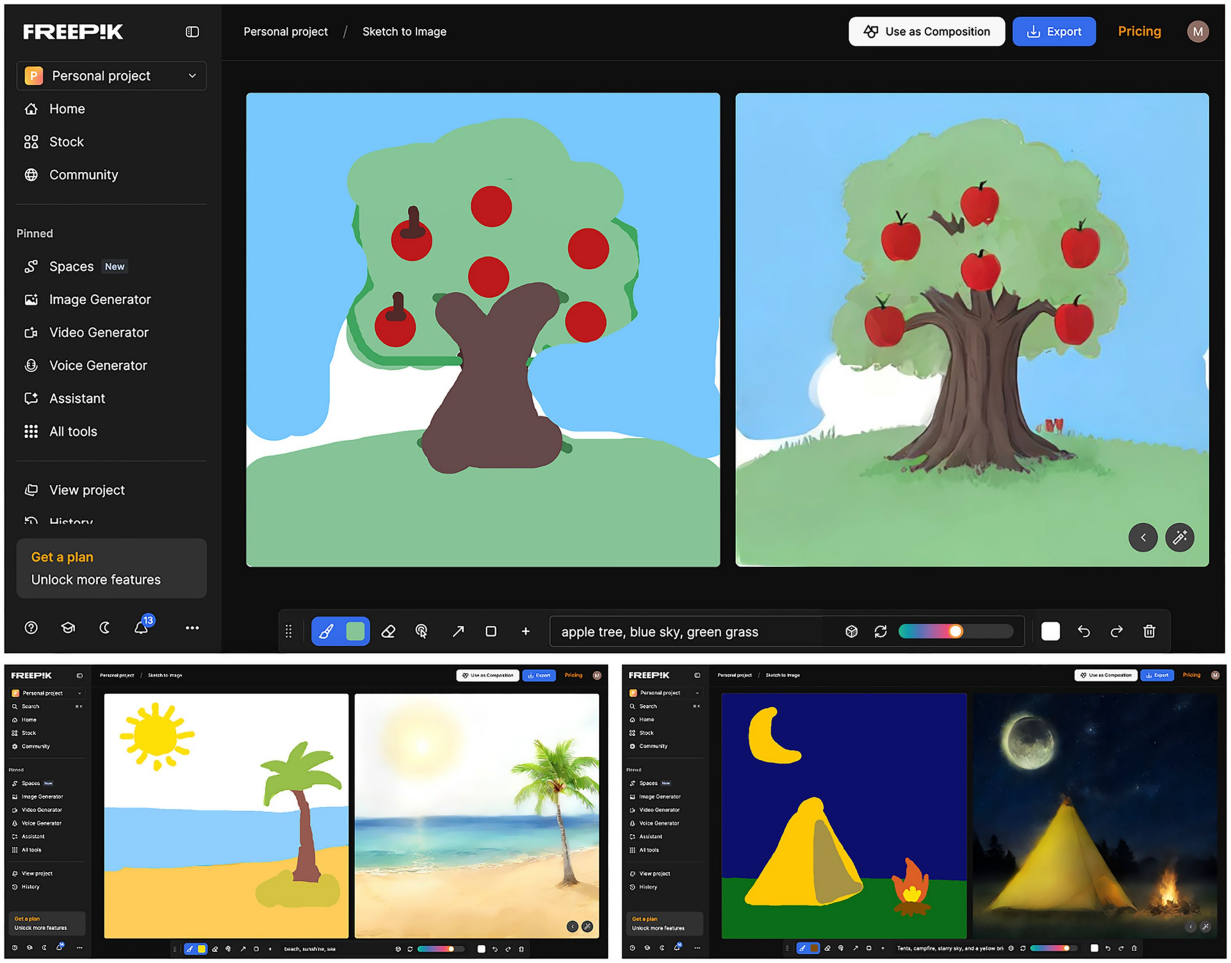
Freepik’s drawing case and interface. Source: Freepik AI platform, https://www.freepik.com/.

Among various AI painting platforms, this study selected Freepik AI as the experimental platform (Figure 2). Freepik AI is a web-based generative art tool that allows users to create images through artificial intelligence based on simple visual inputs and text prompts. Compared with traditional digital drawing software that requires advanced artistic skills, such tools enable users to generate visual content with minimal technical expertise, thereby lowering the barriers to digital art creation. In this study, participants mainly used the “Sketch to Image” function of the platform. This feature allows users to draw simple sketches to outline basic visual elements and spatial structures. After completing the sketch, participants could add short textual descriptions in the input field below the drawing area (e.g., describing objects, colors, or scene elements). These descriptions help the AI system better interpret the user’s creative intention and generate corresponding images. In addition, the platform provides an “Imagination” parameter, which allows users to adjust the degree to which the AI system participates in rendering and completing the final image. By modifying this parameter, participants could control how closely the generated image followed the original sketch versus how much the AI creatively expanded upon the input.

Through this interaction process—drawing simple sketches, supplementing them with textual descriptions, and adjusting the imagination parameter—participants were able to generate AI-assisted artworks. These features were selected because they allow users without professional artistic training to express creative ideas through intuitive interaction, making them particularly suitable for older adults with limited experience in digital art creation.

#### 3.2.2 Questionnaire design and measurement scales

Given that the target participants were older adults, many of whom had limited experience with digital art technologies, a standardized introduction to the AIPT was provided before the questionnaire was administered. All respondents first watched a short demonstration video prepared by the research team, which briefly presented the basic workflow of the platform, including how images could be generated through simple text prompts or sketch inputs. The video served as a unified instructional material to ensure that all participants had a basic understanding of the AIPT. After watching the video, respondents were also given access to the official Freepik AI platform link and were allowed to try the tool voluntarily before completing the questionnaire.

The questionnaire consisted of three sections. The first section contained the standardized introduction described above. The second section collected demographic information, including age, gender, educational background, and experience with digital devices. The third section measured the core research variables. A total of 32 items were used to measure 10 constructs: RA, TR, OQ, IS, PI, HM, PIN, PU, PEOU, and IU. All measurement items were adapted from established studies to ensure content validity and rated on a 7-point Likert scale ranging from 1 (strongly disagree) to 7 (strongly agree).

To improve the clarity and suitability of the questionnaire for older respondents, the draft instrument was reviewed by an interdisciplinary expert panel consisting of scholars in aging and digital technology, a specialist in elderly mental health, and an industry expert in user experience. Based on their feedback, several adjustments were made, including simplifying item wording, revising expressions that might cause cognitive ambiguity for older adults, and optimizing the response scale descriptions. To minimize potential conceptual ambiguity and overlap across all latent variables, all measurement items were carefully distinguished in conceptual connotation and item content. For several experience- and perception-based constructs that are theoretically prone to conceptual confusion, clear definitional boundaries were set in item design. Specifically, OQ refers to evaluations of generated results, HM captures intrinsic enjoyment during engagement, and PU reflects the instrumental value of AIPT for creative goal attainment. The revised questionnaire is presented in Table 2. Ultimately, 50 older adults who volunteered to participate and had basic experience using digital devices were recruited for the pilot test. The pilot results indicated satisfactory reliability and validity, with Cronbach’s *α* values above 0.7 and standardized factor loadings exceeding 0.5 across constructs. Since several measurement items were originally developed in English, a translation and back-translation procedure involving three professional translators was conducted to ensure linguistic accuracy.

**Table 2:** Questionnaire constructs and items.

Variables	Items	Question	Reference
Relative advantage (RA) (3 items)	RA1	Using AIPT allows me to create artworks more easily compared with traditional drawing methods.	Yan et al. (2025), Turner (2007), Rondan-Cataluña et al. (2015)
	RA2	AIPT makes artistic creation more convenient than traditional creating artwork manually.	
	RA3	AIPT allows me to focus more on creative ideas rather than drawing skills.	
Trialability (TR) (3 items)	TR1	Before officially using AIPT, it’s crucial to try it out first.	Tuomi et al. (2023), Benoit (1964), Talantis et al. (2020)
	TR2	Before regularly using AIPT, I can try its operation and explore how it generates the desired artwork.	
	TR3	Before using AIPT, I want to know what it can draw.	
Output quality (OQ) (3 items)	OQ1	I think the artwork generated by AIPT is of very high quality.	DeLone and McLean (2003), Wang (2008), Alzahrani et al. (2019)
	OQ2	The artworks created with AIPT are visually appealing.	
	OQ3	The artworks generated by AIPT are worth keeping or sharing.	
Intergenerational support (IS) (3 items)	IS1	My child or other younger family members encourage me to try using new technology, such as AIPT, for drawing.	Jin and Qu (2025), Jokisch et al. (2022), Serrano (2018)
	IS2	My child or other younger family members are willing to teach me how to use AIPT.	
	IS3	If I encounter problems while using AIPT, my child or other younger family members can help me solve them.	
Peer influence (PI) (3 items)	PI1	If people around me use AIPT, I am more willing to try it.	Venkatesh et al. (2003) Collazo-Castiñeira et al. (2025)
	PI2	When my friends or neighbors find AIPT interesting, I also want to try it.	
	PI3	I am more likely to use AIPT if many people around me use it.	
Hedonistic motivation (HM) (3 items)	HM1	I find the process of creating art with AIPT interesting.	Ramírez-Correa et al. (2018), Venkatesh et al. (2012)
	HM2	I find the process of creating with AIPT exciting.	
	HM3	I enjoy the process of creating art with AIPT.	
Personal innovativeness (PIN) (3 items)	PIN1	I am interested in trying and exploring AIPT as a new creative technology.	Agarwal and Prasad (1998), Hu et al. (2025)
	PIN2	Among people around me, I am usually among the first to try AIPT.	
	PIN3	I am willing to try new AI-based artistic creation tools.	
Perceived usefulness (PU) (4 items)	PU1	Using AIPT helps me express creative ideas more effectively.	Davis (1989), Rispler et al. (2025)
	PU2	Using AIPT saves me time and effort in art creation.	
	PU3	Using AIPT helps me learn more about digital art creation.	
	PU4	Using AIPT helps me generate more creative ideas.	
Perceived ease of use (PEOU) (4 items)	PEOU1	Learning to operate the AIPT was easy for me.	Davis (1989), Jokisch et al. (2022)
	PEOU2	It’s easy to make the AIPT act according to my wishes.	
	PEOU3	I think AIPT is easy to use.	
	PEOU4	I could easily master the use of the AIPT.	
Intention to use (IU) (3 items)	IU1	I intend to use AIPT in the future.	Hwang et al. (2025), Sagnier et al. (2020)
	IU2	I plan to use AIPT for artistic creation.	
	IU3	I will recommend AIPT to others.	

### 3.3 Procedures

Data were collected using convenience sampling combined with mixed online and offline survey methods. For the offline survey, four researchers conducted surveys in elderly residential and activity areas in three cities in China, including Wuxi in Jiangsu Province and Hangzhou and Jiaxing in Zhejiang Province. These sites included city-center residential communities, sports parks, and retirement communities. Before completing the questionnaire, participants were guided to watch AIPT demonstration videos to ensure a basic understanding of the tool and its sketch-to-image functions.

The questionnaire was designed as a self-administered survey and was completed independently by the participants whenever possible. Researchers provided only neutral technical assistance when needed, such as helping participants access the questionnaire link or navigate the online survey interface. They did not explain questionnaire items, interpret questions, suggest answers, infer intentions, or modify responses. Before final submission, the questionnaire was checked only for technical completeness, such as unanswered items. If missing responses were identified, participants were reminded to review the relevant items and decide independently whether to complete them. Any correction or completion was made only by the participants themselves. Questionnaires with unresolved missing responses were treated as invalid and excluded from the final sample. No responses were corrected, supplemented, or replaced by the researchers.

For the online survey, questionnaire links and QR codes were generated through the professional questionnaire platform Wenjuanxing and distributed through mainstream social media channels, including QQ, WeChat group chats, WeChat Moments, and Douyin. The four researchers also shared the questionnaire through older adult activity groups and relevant online communities with the assistance of group administrators. Brief recruitment notices and relevant tags were used to improve the visibility of the questionnaire. To improve data quality, respondents were required to watch the AIPT demonstration video or complete a brief AIPT experience before proceeding to the main questionnaire. The tool experience and questionnaire completion were required to take place on the same day to reduce potential recall bias caused by time intervals. In addition, the Wenjuanxing platform was used to prevent duplicate submissions from the same respondent. A small monetary compensation of 5–10 RMB was provided to acknowledge participants’ time and did not affect the voluntary nature of participation.

All participants completed the questionnaire sections in the same fixed order: standardized AIPT introduction, demographic information, and core research variables. The order was not counterbalanced because the standardized introduction was necessary to ensure that participants had a basic understanding of the AIPT before responding to the measurement items.

### 3.4 Statistical procedures

Data were analyzed using SPSS 27.0, SPSS Amos, and SmartPLS 4.0. Specifically, SPSS 27.0 was used for preliminary data processing, descriptive statistical analysis, and Harman’s single-factor test for common method variance assessment. SPSS Amos was used to conduct the single-factor confirmatory factor analysis (CFA) for further examination of common method variance. SmartPLS 4.0 was used to estimate the partial least squares structural equation model (PLS-SEM), including the assessment of both the measurement model and the structural model (Hair et al., 2019).

Unlike covariance-based structural equation modeling (CB-SEM), PLS-SEM is a variance-based estimation approach that places greater emphasis on the explanatory power of endogenous constructs and the predictive capability of the model, rather than on strict theory testing with an emphasis on overall model fit. The selection of PLS-SEM in this study was based on three main considerations. First, the present study is prediction-oriented, as it aims to explain and predict the mechanism underlying young-old adults’ intention to use AIPTs. Second, the proposed model is relatively complex, involving multiple latent constructs and structural paths. Third, PLS-SEM is more appropriate for simultaneously assessing both the measurement model and the structural model in such complex models, while maximizing the explanatory and predictive power of the endogenous variables. Therefore, PLS-SEM was considered an appropriate analytical approach for this study.

**Figure 3:**
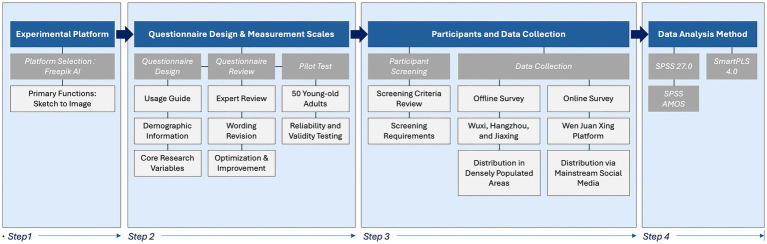
Research process.

The research process is shown in Figure 3.

## 4 Results

Preliminary checks before structural model testing, including common method variance assessment, model fit evaluation, and measurement model assessment, are reported in Supplementary File (Lindell and Whitney, 2001; Craig et al., 2003; Henseler et al., 2015; Akram et al., 2022; Aghimien et al., 2022). These analyses indicated no serious common method variance problem and satisfactory measurement model quality; therefore, the main Results section focuses on the structural model assessment, hypothesis testing, and indirect effects.

The R^2^, adjusted R^2^ and Q^2^ values for each endogenous construct in the model are presented in Table 3. The R^2^ values indicate the explanatory power of the independent variables for the latent variables. Among them, IU (0.340), PEOU (0.337) and PU (0.359) are at a weak to moderate level, indicating that the independent variables have some explanatory power for these latent variables. The adjusted R^2^ values are close to the R^2^ values, indicating that the model has good explanatory stability. Additionally, to assess the model’s predictive relevance, the Stone–Geisser Q^2^ statistic was used through cross-validated redundancy analysis. The results show that all Q^2^ values are greater than 0 (IU is 0.164, PEOU is 0.308, and PU is 0.268), indicating that the model exhibits good predictive relevance for all endogenous variables.

**Table 3:** Values of R^2^ and Q^2^.

Variable	R^2^	Adjusted R^2^	Q^2^
IU	0.340	0.337	0.164
PEOU	0.337	0.327	0.308
PU	0.359	0.347	0.268

To further assess the out-of-sample predictive power of the proposed model, PLSpredict was conducted using a 10-fold cross-validation procedure with 10 repetitions (Shmueli et al., 2019). As shown in Table 4, all Q^2^_predict values for the indicators of the endogenous constructs were greater than zero, indicating that the prediction error comparison was meaningful. Moreover, the RMSE values generated by the PLS-SEM model were lower than those of the linear benchmark model (LM) for all indicators of IU, PEOU, and PU. These findings suggest that the proposed model demonstrates high predictive power.

**Table 4:** PLSpredict results for indicators of endogenous constructs.

Construct	Item	Q^2^_predict	RMSE_PLS	RMSE_LM	RMSE_PLS < RMSE_LM
IU	IU1	0.099	1.515	1.556	Yes
	IU2	0.125	1.526	1.559	Yes
	IU3	0.129	1.548	1.588	Yes
PEOU	PEOU1	0.232	1.512	1.549	Yes
	PEOU2	0.219	1.526	1.565	Yes
	PEOU3	0.198	1.583	1.618	Yes
	PEOU4	0.261	1.499	1.529	Yes
PU	PU1	0.165	1.535	1.555	Yes
	PU2	0.219	1.529	1.554	Yes
	PU3	0.214	1.515	1.534	Yes
	PU4	0.215	1.525	1.552	Yes

PLSpredict was conducted using 10 folds and 10 repetitions. Q^2^_predict values greater than zero indicate predictive relevance. RMSE_PLS and RMSE_LM refer to the root mean squared error generated by the PLS-SEM model and the linear benchmark model, respectively.

To evaluate whether multicollinearity existed among the structural path variables, a Variance Inflation Factor (VIF) test was carried out. The results presented in Table 5 show that all VIF values remained far below the recommended cutoff value of 5.0, and the maximum value observed was 1.752. This suggests that multicollinearity does not pose a serious problem in the model, indicating that the estimated path coefficients are stable and can be interpreted with confidence (Chow et al., 2021).

**Table 5:** Variance inflation factor (VIF) test.

Paths	VIF
HM → PEOU	1.267
HM → PU	1.290
IS → PEOU	1.325
IS → PU	1.404
OQ → PEOU	1.228
OQ → PU	1.231
PEOU → IU	1.752
PEOU → PU	1.509
PI → PEOU	1.270
PI → PU	1.334
PIN → PEOU	1.329
PIN → PU	1.348
PU → IU	1.323
RA → PEOU	1.311
RA → PU	1.328
TR → PEOU	1.264
TR → PU	1.271

**Figure 4:**
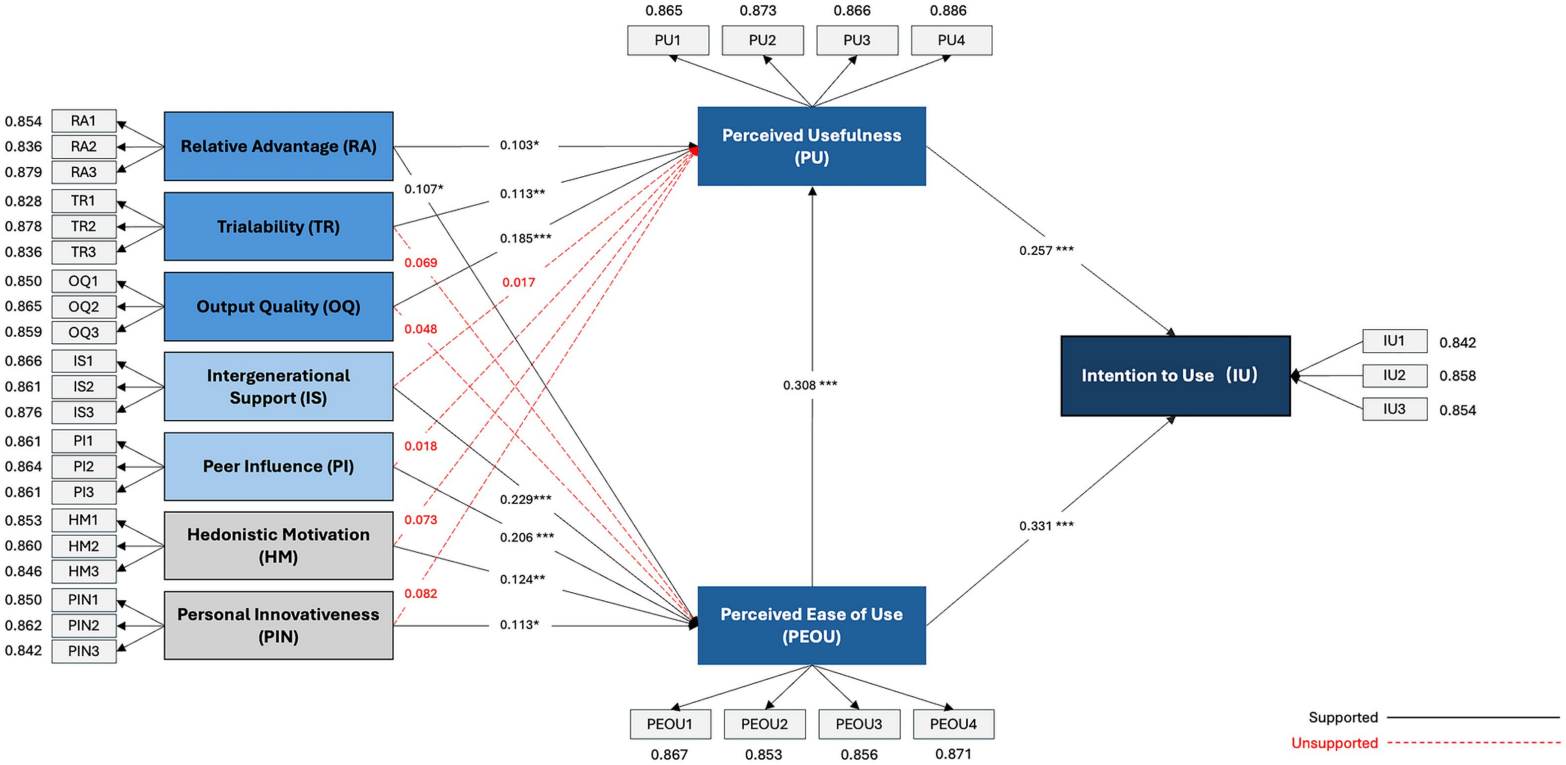
Results of PLS structural model.

Table 6 shows the path coefficients, t-values, *p*-values, and f^2^ effect sizes of the PLS-SEM structural model in this study. To test the significance of the path coefficients, this study used bootstrapping with 5,000 random resamples. The results showed that most of the hypothesized relationships were supported. The detailed results are presented below, and the overall structural model is shown in Figure 4.

**Table 6:** Summary of hypothesis testing.

Hypothesis	Path	*β*	SD	t-statistics	f^2^	*P-*value	Results
H1a	RA → PU	0.103	0.045	2.300	0.012	0.022*	Supported
H1b	RA → PEOU	0.107	0.045	2.375	0.013	0.018*	Supported
H2a	TR → PU	0.113	0.042	2.704	0.016	0.007**	Supported
H2b	TR → PEOU	0.069	0.046	1.509	0.006	0.131	Unsupported
H3a	OQ → PU	0.185	0.042	4.393	0.043	0.000***	Supported
H3b	OQ → PEOU	0.048	0.044	1.085	0.003	0.278	Unsupported
H4a	IS → PU	0.017	0.043	0.400	0.000	0.689	Unsupported
H4b	IS → PEOU	0.229	0.048	4.730	0.060	0.000***	Supported
H5a	PI → PU	0.018	0.041	0.424	0.000	0.671	Unsupported
H5b	PI → PEOU	0.206	0.048	4.251	0.050	0.000***	Supported
H6a	HM → PU	0.073	0.046	1.599	0.006	0.110	Unsupported
H6b	HM → PEOU	0.124	0.044	2.831	0.018	0.005**	Supported
H7a	PIN → PU	0.082	0.044	1.878	0.008	0.060	Unsupported
H7b	PIN → PEOU	0.113	0.047	2.378	0.014	0.017*	Supported
H8	PEOU → PU	0.308	0.048	6.391	0.098	0.000***	Supported
H9	PU → IU	0.257	0.039	6.784	0.079	0.000***	Supported
H10	PEOU → IU	0.331	0.038	10.847	0.191	0.000***	Supported

Bootstrap sample size = 5,000; *p < 0.05, **p < 0.01, ***p < 0.001; path coefficients are estimates from the original sample.

The test results supporting the hypotheses include: H1a (RA → PU) path coefficient is 0.103 (t = 2.300, *p* < 0.05), and H1b (RA → PEOU) path coefficient is 0.107 (t = 2.375, *p* < 0.05), both showing a significant positive relationship, thus supporting the hypotheses; H2a (TR → PU) path coefficient is 0.113 (t = 2.704, *p* < 0.01), showing a significant positive relationship, thus supporting the hypothesis; H3a (OQ → PU) path coefficient is 0.185 (t = 4.393, *p* < 0.001), showing a significant positive relationship, thus supporting the hypothesis. H4b (IS → PEOU) path coefficient is 0.229 (t = 4.730, *p* < 0.001), H5b (PI → PEOU) path coefficient is 0.206 (t = 4.251, *p* < 0.001), both showing a significant positive relationship, thus supporting the hypotheses; H6b (HM → PEOU) path coefficient is 0.124 (t = 2.831, *p* < 0.01), H7b (PIN → PEOU) path coefficient is 0.113 (t = 2.378, *p* < 0.05), both showing a significant positive relationship, thus supporting the hypotheses. In the core path relationships, the path coefficient for H8 (PEOU → PU) was 0.308 (t = 6.391, *p* < 0.001), the path coefficient for H9 (PU → IU) was 0.257 (t = 6.784, *p* < 0.001), and the path coefficient for H10 (PEOU → IU) was 0.331 (t = 10.847, *p* < 0.001). All three relationships were significant and positive, and the corresponding hypotheses were therefore supported, and the path coefficient of H10 was at a relatively high level among all significant paths.

The test results for the unsupported hypotheses included H2b (TR → PEOU) with a path coefficient of 0.069 (t = 1.509, *p* = 0.131), H3b (OQ → PEOU) with a path coefficient of 0.048 (t = 1.085, *p* = 0.278), and H4a (IS → PU) with a path coefficient of 0.017 (t = 0.400, *p* = 0.689). None of these paths showed a significant positive relationship, thus the hypotheses were not supported. Furthermore, H5a (PI → PU) had a path coefficient of 0.018 (t = 0.424, *p* = 0.671), H6a (HM → PU) had a path coefficient of 0.073 (t = 1.599, *p* = 0.110), and H7a (PIN → PU) had a path coefficient of 0.082 (t = 1.878, *p* = 0.060). None of these reached statistical significance, and the corresponding research hypotheses were not supported.

It is also important to interpret the non-significant paths in the proposed model. TR and OQ were not significantly associated with PEOU (TR → PEOU: *β* = 0.069, *p* = 0.131; OQ → PEOU:*β* = 0.048, *p* = 0.278), whereas IS, PI, HM, and PIN were not significantly associated with PU (IS→PU: *β* = 0.017, *p* = 0.689; PI→PU:*β* = 0.018, *p* = 0.671; HM → PU:*β* = 0.073, *p* = 0.110; PIN→PU: *β* = 0.082, *p* = 0.060).

In addition, Cohen’s f^2^ effect size was calculated to assess the substantive contribution of each structural path beyond statistical significance. Consistent with standard PLS-SEM benchmarks, f^2^ values of 0.02, 0.15, and 0.35 indicate small, medium, and large effect sizes, respectively. In this study, the path from PEOU to IU showed a medium effect size (f^2^ = 0.191), indicating that PEOU made the strongest substantive contribution to intention to use among the tested paths. The paths from PEOU to PU (f^2^ = 0.098), PU to IU (f^2^ = 0.079), IS to PEOU (f^2^ = 0.060), PI to PEOU (f^2^ = 0.050), and OQ to PU (f^2^ = 0.043) showed small effect sizes. By comparison, other statistically significant paths, including RA to PU, RA to PEOU, TR to PU, HM to PEOU, and PIN to PEOU, had f^2^ values below the conventional threshold for a small effect, suggesting that their substantive explanatory contributions were relatively limited despite reaching statistical significance. The inclusion of f^2^ values effectively distinguishes statistical significance from substantive impact, improving the rigor and credibility of the findings.

Bootstrapping with 5,000 resamples was performed to test the specific indirect effects. The results in Table 7 show that RA, TR, and OQ showed significant indirect associations with IU through PU. RA, IS, PI, HM, and PIN showed significant indirect associations with IU through PEOU. In addition, the sequential indirect effects of RA, IS, PI, HM, and PIN on IU through PEOU and PU were significant. These results provide formal statistical evidence that PU and PEOU serve as important internal acceptance pathways linking several external factors to young-old adults’ IU. However, not all indirect pathways were significant, suggesting that the mediating roles of PU and PEOU varied across different antecedents.

**Table 7:** Specific indirect effects on IU.

Indirect path	*β*	*Mean*	*STDEV*	t-value	*P*-value	95%CI		Result
						Lower	Upper	
PIN → PU → IU	0.022	0.021	0.012	1.785	0.074	−0.001	0.046	Unsupported
RA → PU → IU	0.027	0.027	0.012	2.215	0.027	0.005	0.053	Supported
TR → PU → IU	0.030	0.030	0.012	2.462	0.014	0.007	0.055	Supported
RA → PEOU → PU → IU	0.009	0.009	0.004	2.076	0.038	0.001	0.018	Supported
PIN → PEOU → PU → IU	0.009	0.009	0.004	2.038	0.042	0.001	0.019	Supported
HM → PEOU → IU	0.050	0.051	0.018	2.750	0.006	0.016	0.087	Supported
IS → PEOU → IU	0.093	0.094	0.023	4.107	0.000	0.051	0.140	Supported
OQ → PEOU → IU	0.020	0.020	0.019	1.053	0.292	−0.015	0.057	Unsupported
PI → PEOU → IU	0.084	0.085	0.021	3.974	0.000	0.045	0.128	Supported
PIN → PEOU → IU	0.046	0.045	0.020	2.321	0.020	0.008	0.085	Supported
RA → PEOU → IU	0.043	0.043	0.019	2.329	0.020	0.007	0.080	Supported
TR → PEOU → PU → IU	0.006	0.006	0.004	1.388	0.165	−0.001	0.014	Unsupported
TR → PEOU → IU	0.028	0.028	0.019	1.493	0.136	−0.007	0.066	Unsupported
PI → PEOU → PU → IU	0.017	0.017	0.006	2.976	0.003	0.007	0.029	Supported
HM → PEOU → PU → IU	0.010	0.010	0.004	2.342	0.019	0.003	0.020	Supported
IS → PEOU → PU → IU	0.018	0.019	0.006	3.218	0.001	0.009	0.031	Supported
OQ → PEOU → PU → IU	0.004	0.004	0.004	1.005	0.315	−0.003	0.012	Unsupported
HM → PU → IU	0.019	0.019	0.013	1.513	0.130	−0.004	0.046	Unsupported
IS → PU → IU	0.005	0.004	0.011	0.400	0.689	−0.018	0.027	Unsupported
OQ → PU → IU	0.049	0.049	0.013	3.738	0.000	0.025	0.075	Supported
PEOU → PU → IU	0.081	0.081	0.018	4.425	0.000	0.048	0.119	Supported
PI → PU → IU	0.005	0.005	0.011	0.428	0.668	−0.017	0.026	Unsupported

β, original sample estimate; STDEV, standard deviation; CI, confidence interval. Bootstrapping was conducted with 5,000 resamples. Indirect effects were considered significant when *p* < 0.05 and the 95% confidence interval did not include zero.

## 5 Discussion

### 5.1 Findings

AIPT provides a promising pathway for older adults to participate in digital creativity and self-expression by lowering the threshold of visual creation. In the context of creative aging, its practical significance depends not only on technological availability, but also on whether older users perceive the tool as useful, easy to use, and worthy of engagement. Against this background, the present study aimed to systematically identify the key factors and pathways associated with young-old adults’ acceptance of AIPT. Grounded in the TAM, a research framework was developed to examine how technological characteristics, social support, and individual motivation are associated with PU, PEOU, and IU. Based on survey data collected from 450 young-old adults and the testing of the proposed structural model, the study yielded the following main findings.

First, regarding Technological characteristics, RA, TR, and OQ were all significantly and positively associated with PU. Among these factors, the association between OQ on PU was the strongest (*β* = 0.185, *p* < 0.001), highlighting the critical role of AI-generated output in shaping users’ evaluation of system usefulness. This finding is consistent with prior research showing that perceived AI output quality is positively associated with PU and behavioral intention (Baroni et al., 2022; Zhou et al., 2025). In the context of AI-powered creative tools, users tend to evaluate usefulness based on whether the generated outputs are meaningful, visually appealing, and relevant to their personal intentions (Y. Liu Y. et al., 2025; Wilson and Burleigh, 2026). Unlike younger users, who may focus more on technical performance or stylistic diversity, older adults are more likely to value the emotional meaning, personal relevance, and expressive potential of AI-generated content. This pattern aligns with findings that PU in AI-supported creative processes is strongly linked to value, efficiency, and creative support (Vaccaro et al., 2024).

In addition, RA (*β* = 0.103, *p* < 0.05) and TR (*β* = 0.113, *p* < 0.01) also showed significant positive associations with PU, which is consistent with the core assumptions of DOI theory. These findings suggest that older adults may perceive AIPT as useful in contexts where the tools are seen as reducing the difficulty of artistic creation and allowing flexible experimentation (Yildirim et al., 2023). Similar results have been reported in studies on generative AI tools, where usability, perceived benefits, and experiential exploration are positively associated with PU (Kim and Kim, 2025).

With respect to PEOU, only RA showed a significant positive association, whereas TR and OQ were not significant predictors. This suggests that young-old adults’ perceptions of ease-of-use depend more on whether the technology simplifies the creative process than on the availability of trial opportunities or the quality of the generated outputs (Akverdi and Baykal, 2024). In practical terms, even when a tool can produce high-quality images, users may still regard it as difficult to use if the interface design or interaction process is overly complex (Yildirim et al., 2023). This finding underscores the importance of usability-oriented design in the development of AI tools for young-old adults, highlighting the need for clear interaction logic, intuitive operation, and immediate feedback mechanisms.

Second, regarding social support factors, IS and PI were both significantly and positively associated with PEOU (IS → PEOU: *β* = 0.229, *p* < 0.001; PI → PEOU: *β* = 0.206, *p* < 0.001), whereas their associations with PU were not significant. These findings suggest that social support primarily facilitates the learning and operational dimensions of technology use (Zhou et al., 2024), rather than directly shaping perceptions of usefulness. Guidance from family members and experience sharing among peers may help older adults become more familiar with the interface and reduce uncertainty during operation (Lai et al., 2025), thereby strengthening their perceptions of ease of use. However, the PU of AIPT appears to depend more on users’ direct experiences in the creative process, particularly whether the tool effectively supports their personal creative intentions (Li et al., 2021). The significant associations of IS and PI with PEOU further suggest that social support mainly helps older adults reduce perceived operational difficulty rather than directly changing their evaluation of usefulness.

The cultural context of the Chinese sample may further contextualize the importance of social support in relation PEOU. In Chinese urban communities, older adults’ digital technology use is often embedded in family-centered and community-based social networks. Adult children and younger family members may provide not only operational guidance but also emotional reassurance and technology-related encouragement, reflecting the continuing influence of intergenerational responsibility and filial norms. Similarly, PI may provide social proof and normalize difficulties in learning unfamiliar digital tools, allowing older adults to gain confidence through shared experiences. These mechanisms are more directly related to PEOU because they help older adults feel that they can operate AIPT, rather than directly convincing them of its usefulness for personal creative goals. Therefore, the associations of IS and PI should be interpreted in relation to the collectivist, family-oriented, and community-based context of the Chinese sample. Caution is needed when generalizing these findings to cultural contexts with different family structures, IS norms, and community-based learning resources, because the role of family and peer support in older adults’ technology adoption may vary across contexts.

Third, in terms of individual motivation, HM and PIN both showed significant positive associations with PEOU (HM → PEOU: *β* = 0.124, *p* < 0.01; PIN → PEOU: *β* = 0.113, *p* < 0.05), while neither showed a significant association with PU. This finding is consistent with prior studies indicating that intrinsic motivational factors such as enjoyment and innovativeness are more closely associated with users’ perceptions of ease of interaction rather than their evaluation of functional utility (Li et al., 2025; Liu M. et al., 2025). One possible explanation is that HM encourages users to actively explore system functionalities, thereby reducing perceived difficulty caused by unfamiliarity, whereas PIN increases tolerance toward early-stage operational errors and uncertainty (Yang et al., 2023).

At the same time, the non-significant associations with PU suggest that these individual traits do not directly determine whether older users perceive the tool as practically valuable. Instead, PU appears to be shaped through actual usage experiences and task-related outcomes, such as whether the tool effectively supports users’ creative goals (Bianchi and Saleh, 2024). Overall, this pattern implies that intrinsic motivational factors are primarily associated with engagement with the interaction process, particularly at the stage of usability perception, rather than directly shaping evaluations of functional usefulness (Saare et al., 2019).

In addition, the non-significant paths provide further insight into the differentiated belief-formation pattern of AIPT acceptance among young-old adults. TR and OQ were not significantly associated with PEOU, suggesting that trial opportunities and high-quality outputs may be more closely related to exploratory engagement and perceived creative value than to perceived operational ease. In other words, even if older users have opportunities to try the tool or perceive its outputs as visually satisfactory, these factors do not necessarily correspond to lower perceived difficulty in sketch-to-image interaction. Similarly, IS, PI, HM, and PIN were not significantly associated with PU, indicating that social encouragement, peer experience, enjoyment, and openness to innovation may be more closely related to confidence, reduced uncertainty, and willingness to explore unfamiliar AI tools than to direct evaluations of usefulness. Therefore, these unsupported hypotheses should not be interpreted simply as failed paths.

Finally, the core pathways of the TAM were fully supported. PEOU not only had a direct positively associated with IU (*β* = 0.331, *p* < 0.001), but also was indirectly associated with IU through PU (PEOU→PU: *β* = 0.308, *p* < 0.001; PU → IU: *β* = 0.257, *p* < 0.001). Notably, the direct association of PEOU on IU was stronger than that of PU, which differs from the pattern commonly reported in studies of younger users (Davis, 1989; Venkatesh et al., 2003). For older users, whether a tool is easy to use appears to be a more decisive factor in shaping usage intention than whether it is useful (Yau and Shen, 2025). A likely reason is that because elderly groups have limited energy for learning new technologies, and even if they recognize the tool’s usefulness, they may abandon it due to perceived operational difficulties (Shojaei et al., 2024). This finding highlights the central importance of usability design in AIPT for older adults and provides an important theoretical basis for the optimizing of age-friendly digital products.

### 5.2 Theoretical and practical contributions

From a theoretical perspective, this study advances the TAM through a context-driven theoretical refinement in the domain of AI-assisted creative aging, rather than serving as a mere contextual application. While prior extensions such as TAM2 and UTAUT have incorporated additional determinants (e.g., social influence, facilitating conditions, and performance expectations), they remain largely grounded in utilitarian and task-oriented technology use contexts. By contrast, this study repositions TAM within a later-life creative engagement context, where technology adoption is associated not only with efficiency or task completion, but also with creative participation, self-expression, exploratory engagement, and digital inclusion. In doing so, the study extends the explanatory scope of TAM from performance-oriented usage to meaning-oriented and experience-driven interaction, thereby broadening its applicability to non-utilitarian and experience-centric domains.

More importantly, this study contributes a mechanism-level refinement by conceptualizing technology adoption as a multidimensional belief-formation process shaped by technological, social, and motivational conditions. The findings reveal a differentiated dual-path structure: technological characteristics were generally more closely associated with PU, representing a value-oriented evaluation pathway; in contrast, social support and individual motivational factors were more closely associated with PEOU, reflecting a capability-oriented pathway related to reduced uncertainty and stronger operational confidence. More specifically, TR and OQ were mainly associated with PU, while RA was associated with both PU and PEOU, suggesting that RA may serve as a bridging technological belief that connects value-oriented and capability-oriented evaluations.

This distinction demonstrates that AIPT adoption among young-old adults is not driven by a single linear mechanism, but emerges from the interaction between value-based and capability-based beliefs across domains. Furthermore, the findings suggest that, in this context, capability-related beliefs (PEOU) may play a more foundational role, as older users must first establish a sense of control before fully recognizing the creative value of the technology. This insight refines the theoretical understanding of belief prioritization in later-life technology adoption and highlights a boundary condition under which PEOU may precede PU, thereby offering a nuanced extension to the conventional TAM assumption regarding the primacy of PU.

From a practical perspective, the findings offer implications for the design and promotion of AIPT among young-old adults. Given that PEOU showed a stronger association with IU than PU, the design of AIPT should prioritize reducing operational uncertainty and enhancing older adults’ sense of control over the creative process. For developers, age-friendly design should not only focus on interface accessibility, but also on simplifying the sketch-based interaction workflow. Specifically, platforms could adopt clear interface hierarchies, legible visual elements, explicitly labeled functions, one-click generation, and accessible undo, redraw, and retry operations. More importantly, onboarding mechanisms should serve as sketch-based creative scaffolding, including beginner modes, step-by-step visual tutorials, sample sketches, contour templates, and guidance on line depiction, shape construction, composition, and object arrangement. Such support may help older adults gradually move from guided imitation to independent creative exploration.

Furthermore, AI explainability should be framed as creative feedback rather than technical algorithmic explanation. Older adults may not require detailed algorithmic explanations, but they need to understand how their hand-drawn sketches shape AI-generated images and what concrete adjustments can improve creative outcomes. Accordingly, AIPT platforms should provide straightforward visual feedback on how line clarity, object boundaries, compositional structure, and stylistic choices affect output quality, along with targeted revision suggestions when generated results do not meet users’ expectations. In this way, explainability can help transform AI generation from an unpredictable process into a more understandable and controllable form of human–AI co-creation.

For family members, particularly adult children, intergenerational guidance should emphasize explanation, demonstration, encouragement, and supportive feedback, while avoiding replacing older adults’ own creative decision-making. Such support may reduce operational anxiety and uncertainty and help older users build confidence in using AIPT independently. For community organizations, creative workshops, collective art activities, and socially embedded learning opportunities can provide an important environment for peer learning and sustained engagement. Community activities structured around demonstration, guided practice, independent creation, and experience sharing may help alleviate technology anxiety, normalize initial learning difficulties, and create a supportive atmosphere for older adults’ continued participation in AI-assisted creative activities.

### 5.3 Research limitations and future research directions

This study has several limitations. First, the cross-sectional design captured participants’ perceptions and intention to use AIPT at a single point in time. Although the proposed model was empirically supported, this design limits causal inference and cannot reveal changes in older adults’ AIPT acceptance over time or its association with sustained use behavior. Second, the sample mainly consisted of Chinese urban young-old adults aged 60–69 with basic digital skills who could complete the questionnaire independently or with limited assistance. This sampling feature may introduce selection bias and limit external validity. Participants with higher digital familiarity may perceive fewer operational barriers, experience less uncertainty, and feel more confident when interacting with AI tools, which may correspond to higher reported intention to use AIPT than among older adults with lower digital literacy. Therefore, the findings may better reflect digitally connected young-old adults than digitally disadvantaged older adults and should be generalized with caution to older adults with lower digital literacy, limited access to digital technologies, rural older adults, old-old adults, digitally excluded populations, or cultural contexts with different family structures and social support systems. Third, this study used only Freepik AI as the representative AIPT platform, whereas other platforms may differ in interface design, interaction logic, ease of operation, and output quality. Finally, because this study adopted a quantitative approach and focused on technology acceptance variables, it did not fully capture older adults’ deeper cognitive, emotional, and subjective experiences in AI-assisted creative activities.

Future research could extend this study in several ways. First, longitudinal or experimental designs could be adopted to examine changes in older adults’ AIPT acceptance over time and further clarify how intention to use is associated with sustained use behavior. Second, future studies could adopt stratified or quota sampling to include older adults with different age cohorts, digital literacy levels, education levels, urban–rural backgrounds, and prior AI experience. Comparative analyses between young-old and old-old adults, as well as multi-group analyses based on digital competence, could help examine whether the proposed model remains valid across different later-life populations. Third, cross-cultural comparisons could be conducted to examine whether the roles of IS and PI remain consistent across cultural contexts with different family structures, filial norms, community resources, and social support systems. Fourth, future research could compare multiple AIPT platforms across different use contexts to test the robustness and applicability of the proposed model under different technological conditions. Finally, qualitative methods and theoretical perspectives such as cognitive aging and emotional aging could be incorporated to provide a deeper understanding of how older adults perceive, experience, and adopt AIPT.

## 6 Conclusion

This study examined factors associated with young-old adults’ intention to use AIPT by integrating the TAM with technological characteristics, social support, and individual motivational factors. Using Freepik AI as a representative case and applying PLS-SEM analysis, the study examined how young-old adults perceive and accept AIPT. The findings showed that both PEOU and PU were positively associated with IU, with PEOU showing a stronger association. Among the technological characteristics, TR and OQ were mainly associated with PU, while RA was associated with both PU and PEOU. Social support factors, including IS and PI, and individual motivational factors, including HM and PIN, were significantly associated with PEOU but not with PU. Overall, this study suggests that AIPT acceptance among young-old adults is associated with technological conditions, social context, and personal motivation. By identifying these differentiated pathways, the study provides a clearer understanding of how AIPT may be introduced to older users in a more age-friendly way and offers an empirical basis for further research on AI-supported creative engagement in aging populations.

## Data Availability

The original contributions presented in the study are included in the article/Supplementary material, further inquiries can be directed to the corresponding author.

## References

[ref1] Adams-Price C. E. Morse L. W. (2025). Creativity, aging, context and culture: reimagining creativity in everyday life in older adults. Possibil. Stud. Soc. 3, 499–523. doi: 10.1177/27538699241235247

[ref2] Agarwal R. Prasad J. (1998). A conceptual and operational definition of personal innovativeness in the domain of information technology. Inf. Syst. Res. 9, 204–215. doi: 10.1287/isre.9.2.204, 19642375

[ref3] Aghimien D. Ikuabe M. Aghimien L. M. Aigbavboa C. Ngcobo N. Yankah J. (2022). PLS-SEM assessment of the impediments of robotics and automation deployment for effective construction health and safety. J. Facil. Manag. 22, 458–478. doi: 10.1108/JFM-04-2022-0037, 35579975

[ref4] Akram K. Saeed A. Bresciani S. Rehman S. U. Ferraris A. (2022). Factors affecting environmental performance during the COVID-19 period in the leather industry: a moderated-mediation approach. JOC 14, 5–22. doi: 10.7441/joc.2022.01.01

[ref5] Akverdi C. Baykal G. E. (2024). Generative AI tools in design fields: opportunities and challenges in the ideation process. ACM. 29, 1–5. doi: 10.1145/3677045.3685445

[ref6] Ali I. Warraich N. F. Butt K. (2025). Acceptance and use of artificial intelligence and AI-based applications in education: a meta-analysis and future direction. Inf. Dev. 41, 859–874. doi: 10.1177/02666669241257206

[ref7] Alzahrani A. I. Mahmud I. Ramayah T. Alfarraj O. Alalwan N. (2019). Modelling digital library success using the DeLone and McLean information system success model. J. Librariansh. Inf. Sci. 51, 291–306. doi: 10.1177/0961000617726123

[ref8] Baroni I. Calegari G. R. Scandolari D. Celino I. (2022). AI-TAM: a model to investigate user acceptance and collaborative intention in human-in-the-loop AI applications. Hum. Comput. 9, 1–21. doi: 10.15346/hc.v9i1.134

[ref9] Bell C. Fausset C. Farmer S. Nguyen J. Harley L. Fain W. B., (2013). Examining social media use among older adults, in: Proceedings of the 24th ACM Conference on Hypertext and Social Media. Presented at the HT ‘13: 24th ACM Conference on Hypertext and Social Media, ACM, Paris France, pp. 158–163.

[ref10] Benoit O. (1964). Review of diffusion of innovations. Rev. Fr. Sociol. 5, 216–218. doi: 10.2307/3319808

[ref11] Bhowmick P. (2023). Peer-based check-in support and personal AGENCY in tangible interface design for reducing isolation in older adults. Innov. Aging 7:1381. doi: 10.1093/geroni/igad104.1381

[ref12] Bianchi C. Saleh M. A. (2024). Investigating the drivers of E-commerce continuance intentions among older consumers in Latin America. J. Internet Commer. 23, 384–413. doi: 10.1080/15332861.2024.2424141

[ref13] Binyamin S. S. Zafar B. A. (2021). Proposing a mobile apps acceptance model for users in the health area: a systematic literature review and meta-analysis. Health Informatics J. 27:1460458220976737. doi: 10.1177/1460458220976737, 33438494

[ref14] Bomba F. De Angeli A. (2025). Agency and authorship in AI art: transformational practices for epistemic troubles. Int. J. Hum.-Comput. Stud. 205:103652. doi: 10.1016/j.ijhcs.2025.103652

[ref15] Cho E. Chang W. (2024). Facilitating arts participation for creative ageing: an action research in South Korea. Ageing Soc. 44, 1031–1050. doi: 10.1017/S0144686X22000551

[ref16] Chow T. C. Zailani S. Rahman M. K. Qiannan Z. Bhuiyan M. A. Patwary A. K. (2021). Impact of sustainable project management on project plan and project success of the manufacturing firm: structural model assessment. PLoS One 16:e0259819. doi: 10.1371/journal.pone.0259819, 34818357 PMC8612515

[ref17] Collazo-Castiñeira P. Rodríguez-Rey R. Cruz-Jentoft A. J. Allouch S. B. Eglseer D. Schoufour J. . (2025). Tailoring mHealth for healthy aging: focus group study with retirement-age adults. JMIR Mhealth Uhealth 13:51. doi: 10.2196/70051, 41397687 PMC12750076

[ref18] Craig C. L. Marshall A. L. Sjöström M. Bauman A. E. Booth M. L. Ainsworth B. E. . (2003). International physical activity questionnaire: 12-country reliability and validity. Med. Sci. Sports Exerc. 35, 1381–1395. doi: 10.1249/01.MSS.0000078924.61453.FB, 12900694

[ref19] Damilig A. D. (2025). Assessing the determinants of faculty acceptance of generative AI in research practices. Int. J. For Multidiscip. Res. 7:724. doi: 10.36948/ijfmr.2025.v07i04.51724

[ref20] Davis F. D. (1989). Perceived usefulness, perceived ease of use, and user acceptance of information technology. MIS Q. 13, 319–340. doi: 10.2307/249008

[ref21] DeLone W. H. McLean E. R. (2003). The DeLone and McLean model of information systems success: a ten-year update. J. Manag. Inf. Syst. 19, 9–30. doi: 10.1080/07421222.2003.11045748, 37339054

[ref22] Du Y. Li T. Gao C. (2023). Why do designers in various fields have different attitude and behavioral intention towards AI painting tools? An extended UTAUT model. Proc. Comput. Sci. 221, 1519–1526. doi: 10.1016/j.procs.2023.08.010

[ref23] Edwards L. Owen-Booth B. (2021). An exploration of engagement in community based creative activities as an occupation for older adults. Ir. J. Occup. Ther. 49, 51–57. doi: 10.1108/IJOT-05-2020-0009

[ref24] Evans S. C. Bray J. Garabedian C. (2022). Supporting creative ageing through the arts: the impacts and implementation of a creative arts programme for older people. WWOP 26, 22–30. doi: 10.1108/WWOP-03-2021-0014

[ref25] Fisher B. J. Specht D. K. (1999). Successful aging and creativity in later life. J. Aging Stud. 13, 457–472. doi: 10.1016/S0890-4065(99)00021-3

[ref26] Hair J. F. Risher J. J. Sarstedt M. Ringle C. M. (2019). When to use and how to report the results of PLS-SEM | European business review | emerald publishing. Eur. Bus. Rev. 1:203. doi: 10.1108/EBR-11-2018-0203

[ref27] Hajek A. Zwar L. Gyasi R. M. Yon D. K. Pengpid S. Peltzer K. . (2025). Association of using AI tools for personal conversation with social disconnectedness outcomes. J. Public Health. doi: 10.1007/s10389-025-02554-6

[ref28] Hamadeh A. H. Nouraldeen R. M. Mahboub R. M. Hashem M. S. (2025). Auditors’ intention to use Blockchain technology and TAM3: the moderating role of age. Admin. Sci. 15:61. doi: 10.3390/admsci15020061

[ref29] Han S. Yang H. (2018). Understanding adoption of intelligent personal assistants: a parasocial relationship perspective. IMDS 118, 618–636. doi: 10.1108/IMDS-05-2017-0214

[ref30] Hao X. Cui Y. (2025) Smart museums, smarter experiences: exploring the impact of GenAI usability on digital heritage enjoyment, in: Proceedings of the 2025 2nd International Conference on Digital Systems and Design Innovation, ICDSDI ‘25. Association for Computing Machinery, New York, pp. 107–117.

[ref31] Harris M. T. Rogers W. A. (2023) Developing a Healthcare Technology Acceptance Model (H-TAM) for Older Adults with Hypertension. Ageing Soc., 43, 814–834. doi: 10.1017/S0144686X21001069 PMC10062492 37007645

[ref32] Hennelly N. Cooney A. Houghton C. O’Shea E. (2019) Personhood and Dementia Care: A Qualitative Evidence Synthesis of the Perspectives of People With Dementia. Oxford, United Kingdom: Oxford University Press, on behalf of The Gerontological Society of America. 10.1093/geront/gnz159 31854441

[ref33] Henseler J. Ringle C. M. Sarstedt M. (2015). A new criterion for assessing discriminant validity in variance-based structural equation modeling. J. Acad. Mark. Sci. 43, 115–111. doi: 10.1007/s11747-014-0403-8

[ref34] Hu L. Chen M. Ding N. (2025). Factors influencing digital media designers’ subscription to premium versions of AI drawing tools through a mixed methods study. Sci. Rep. 15:15994. doi: 10.1038/s41598-025-99924-7, 40341292 PMC12062495

[ref35] Huang B. Yang Y. (2025). Revisiting the technology acceptance models: a critical evaluation and their educational applications. Asian J. Educ. Soc. Stud. 51, 80–91. doi: 10.9734/ajess/2025/v51i31810

[ref36] Hwang S. Byun H. Yi E.-S. (2025). Understanding older adults’ intention to adopt digital leisure services: the role of psychosocial factors and AI-based prediction models. Healthcare 13:785. doi: 10.3390/healthcare13070785, 40218082 PMC11988560

[ref37] Iqbal S. Fischl C. Asai R. (2025). Older persons’ social participation, health and well-being through digital engagement. Activit. Adapt. Aging 49:299. doi: 10.1080/01924788.2025.2512299

[ref38] Jeong T. Hwang G. Kim D. Y. (2025). A generative AI framework for cognitive intervention in older adults: an integrated engineering design and clinical protocol. Healthcare 13:225. doi: 10.3390/healthcare13243225, 41464294 PMC12732633

[ref39] Jeong N.-Y. Yoo Y. (2007) A study of adopting Warshaw’s purchase intention model in Mobile-RFID services and on moderating effect of personal innovativeness, in: PICMET ‘07–2007 Portland International Conference on Management of Engineering & Technology. Presented at the PICMET ‘07–2007 Portland International Conference on Management of Engineering & Technology, pp. 2932–2939.

[ref40] Jin H. Qu Y. (2025). Association between intergenerational support, technology perception and trust, and intention to seek medical care on the internet among Chinese older adults: cross-sectional questionnaire study. J. Med. Internet Res. 27:65. doi: 10.2196/65065, 39761564 PMC11747539

[ref41] Jokisch M. R. Schmidt L. I. Doh M. (2022). Acceptance of digital health services among older adults: findings on perceived usefulness, self-efficacy, privacy concerns, ICT knowledge, and support seeking. Front. Public Health 10:1073756. doi: 10.3389/fpubh.2022.1073756, 36582385 PMC9792847

[ref42] Kaimal G. Carroll-Haskins K. Mensinger J. L. Dieterich-Hartwell R. M. Manders E. Levin W. P. (2019). Outcomes of art therapy and coloring for professional and informal caregivers of patients in a radiation oncology unit: a mixed methods pilot study. Eur. J. Oncol. Nurs. 42, 153–161. doi: 10.1016/j.ejon.2019.08.006, 31557665

[ref43] Kim J. Kim H. (2025). User perceptions and intentions to continue using generative AI tools in the design process. Edelweiss Appl. Sci. Technol. 9, 24–31. doi: 10.55214/25768484.v9i4.5933

[ref44] Lai F. H.-Y. Yip B. C.-B. Hai E. Y.-K. Darling C. Man D. W.-K. (2025). Acceptability and feasibility in using gamified mobile application for working memory training in older adults. Health Soc. Care Community 2025:547. doi: 10.1155/hsc/9925547

[ref45] Lee J.-H. Lee C.-F. (2019). Extension of TAM by perceived interactivity to understand usage behaviors on ACG social media sites. Sustainability 11:5723. doi: 10.3390/su11205723

[ref46] Li W. (2025). A study on factors influencing designers’ behavioral intention in using AI-generated content for assisted design: perceived anxiety, perceived risk, and UTAUT. Int. J. Hum. Comput. Interact. 41, 1064–1077. doi: 10.1080/10447318.2024.2310354

[ref47] Li W. Shen S. Yang J. Tang Q. (2021). Internet-based medical service use and Eudaimonic well-being of urban older adults: a peer support and technology acceptance model. Int. J. Environ. Res. Public Health 18:62. doi: 10.3390/ijerph182212062, 34831817 PMC8618015

[ref48] Li Y. Siek H. L. Guo J. (2025). An empirical study on the use behavior towards AI painting tools based on TAM3 model. Sci. Rep. 15:40533. doi: 10.1038/s41598-025-24405-w, 41254140 PMC12627507

[ref49] Lindell M. K. Whitney D. J. (2001). Accounting for common method variance in cross-sectional research designs. J. Appl. Psychol. 86, 114–121. doi: 10.1037/0021-9010.86.1.114, 11302223

[ref50] Linden T. Yuan K. Mendoza A. (2025). The potential and challenges of integrating generative AI in higher education as perceived by teaching staff: a phenomenological study. Inf. Syst. Educ. J. 23:578. doi: 10.62273/IENP8578

[ref51] Liu Z. Agudamu Bu T. Akpinar S. Jabucanin B. (2022). The association between the China’s economic development and the passing rate of national physical fitness standards for elderly people aged 60–69 from 2000 to 2020. Front. Public Health 10:857691. doi: 10.3389/fpubh.2022.857691 35359759 PMC8961805

[ref52] Liu M. Wang C. Hu J. (2025). Understanding older adults’ adoption of facial recognition payment: an integrated model of TAM and UXT. PLoS One 20:e0325291. doi: 10.1371/journal.pone.0325291, 40638598 PMC12244727

[ref53] Liu Y. Yang Y. Xu H. (2025). From humans to AI: understanding why AI is perceived as the preferred co-creation partner. Front. Psychol. 16:1695532. doi: 10.3389/fpsyg.2025.1695532, 41446615 PMC12722866

[ref54] Löfgren L. (2021). Older adults’ experiences of maintaining social participation: creating opportunities and striving to adapt to changing situations. Scand. J. Occup. Ther. 29, 587–597. doi: 10.1080/11038128.2021.1974550, 34499845

[ref55] Lu Q. Rong B. Meng X. Luo X. Chen Q. Xi G. . (2025). WeChat adoption among older adults and urban-rural differences in China. Int. J. Hum. Comput. Interact. 41, 5591–5606. doi: 10.1080/10447318.2024.2364475

[ref56] Ma Z. Gao Q. Yang M. (2023). Adoption of wearable devices by older people: changes in use behaviors and user experiences. Int. J. Hum. Comput. Interact. 39, 964–987. doi: 10.1080/10447318.2022.2083573

[ref57] Ma X. Li Y. Yang Y. (2025) Understanding designers’ adoption of AI tools: a combined perspective of technology acceptance model and innovation diffusion theory, in: Proceedings of the 2025 2nd International Conference on Digital Society and Artificial Intelligence, DSAI ‘25. Association for Computing Machinery, New York, pp. 63–70.

[ref58] Malatji W. R. Eck R. V. Zuva T. (2020). Understanding the usage, modifications, limitations and criticisms of technology acceptance model (TAM). Adv. Sci. Technol. Eng. Syst. J. 5, 113–117. doi: 10.25046/aj050612

[ref59] Martín-García A. V. Redolat R. Pinazo-Hernandis S. (2021). Factors influencing intention to technological use in older adults. The TAM model aplication - Antonio V. Martín-García, Rosa Redolat, Sacramento Pinazo-Hernandis, 2022. Res. Aging. 44, 573–588. doi: 10.1177/01640275211063797 34962846

[ref60] Mei Y. (2024). Exploring the mechanisms driving elderly Fintech engagement: the role of social influence and the elderly’s digital literacy. Front. Psychol. 15:1420147. doi: 10.3389/fpsyg.2024.1420147, 38974106 PMC11224538

[ref61] Pang W. Y. J. Cheng L. (2023) Acceptance of Gamified Virtual Reality Environments by Older Adults. Educational Gerontology. Abingdon, United Kingdom: Routledge, Taylor & Francis Group. 49, 831–841. doi: 10.1080/03601277.2023.2166262

[ref62] Patil D. (2024) ChatGPT and Similar Generative Artificial Intelligence in Art, Music, and Literature Industries: Applications and Ethical Challenges. Rochester, NY, United States: Social Science Research Network (SSRN), Elsevier.

[ref63] Ramírez-Correa P. E. Rondán-Cataluña F. J. Arenas-Gaitán J. (2018). Student information system satisfaction in higher education: the role of visual aesthetics. Kybernetes 47, 1604–1622. doi: 10.1108/K-08-2017-0297, 35579975

[ref64] Reynolds F. (2010). ‘Colour and communion’: exploring the influences of visual art-making as a leisure activity on older women’s subjective well-being. J. Aging Stud. 24, 135–143. doi: 10.1016/j.jaging.2008.10.004

[ref65] Rispler C. Mashiach-Eizenberg M. Yakov G. (2025). Understanding students’ perceptions of generative AI: implications for pedagogy and graduate employability. J. Teach. Learn. Grad. Employ. 16, 145–170. doi: 10.21153/jtlge2025vol16no1art2084

[ref66] Rondan-Cataluña F. J. Arenas-Gaitán J. Ramírez-Correa P. E. (2015). A comparison of the different versions of popular technology acceptance models: a non-linear perspective. Kybernetes 44, 788–805. doi: 10.1108/K-09-2014-0184

[ref67] Russo D. (2024). Navigating the complexity of generative AI adoption in software engineering. ACM Trans. Softw. Eng. Methodol. 33, 1–50. doi: 10.1145/3652154

[ref68] Saare M. A. Hussain A. Yue W. S. (2019). Conceptualizing mobile health application use intention and adoption among Iraqian older adults: from the perspective of expanded technology acceptance model. Int. J. Interact. Mobile Technol. 13, 28–41. doi: 10.3991/ijim.v13i10.11285

[ref69] Sagnier C. Loup-Escande E. Lourdeaux D. Thouvenin I. Valléry G. (2020). User acceptance of virtual reality: an extended technology acceptance model. Int. J. Hum. Comput. Interact. 36, 993–1007. doi: 10.1080/10447318.2019.1708612

[ref70] Serrano I. (2018) Evaluating the Social and Technological Benefits of an Intergeneration. Kingston, RI, United States: University of Rhode Island.

[ref71] Shen X. Mo X. Xia T. (2024). Exploring the attitude and use of GenAI-image among art and design college students based on TAM and SDT. Interact. Learn. Environ. 1–18, 1–18. doi: 10.1080/10494820.2024.2365959, 37339054

[ref72] Shmueli G. Sarstedt M. Hair J. F. Cheah J.-H. Ting H. Vaithilingam S. . (2019). Predictive model assessment in PLS-SEM: guidelines for using PLSpredict. Eur. J. Mark. 53, 2322–2347. doi: 10.1108/EJM-02-2019-0189

[ref73] Shojaei F. Shojaei F. Osorio Torres J. Shih P. C. (2024). Insights from art therapists on using AI-generated art in art therapy: mixed methods study. JMIR Form. Res. 8:e63038. doi: 10.2196/63038, 39631077 PMC11634044

[ref74] Siette J. Guion J. Ija K. Strut P. Porte M. Savage G. . (2024). Development of a new computer simulated environment to screen cognition: assessing the feasibility and acceptability of leaf café in younger and older adults. BMC Med. Inform. Decis. Mak. 24:79. doi: 10.1186/s12911-024-02478-3 38504250 PMC10949698

[ref75] Syed-Abdul S. Malwade S. Nursetyo A. A. Sood M. Bhatia M. Barsasella D. . (2019). Virtual reality among the elderly: a usefulness and acceptance study from Taiwan. BMC Geriatr. 19:18. doi: 10.1186/s12877-019-1218-8, 31426766 PMC6699111

[ref76] Talantis S. Shin Y. H. Severt K. (2020). Conference mobile application: participant acceptance and the correlation with overall event satisfaction utilizing the technology acceptance model (TAM). J. Convent. Event Tour. 21, 100–122. doi: 10.1080/15470148.2020.1719949

[ref77] Tankelevitch L. Kewenig V. Simkute A. Scott A. E. Sarkar A. Sellen A. ., (2024) The metacognitive demands and opportunities of generative AI, in Proceedings of the 2024 CHI Conference on Human Factors in Computing Systems, ACM Conferences. pp. 1–24.

[ref78] Tuomi A. Moreira Kares E. Zainal Abidin H. (2023). Digital cultural tourism: older adults’ acceptance and use of digital cultural tourism services. Scand. J. Hosp. Tour. 23, 226–247. doi: 10.1080/15022250.2023.2256698

[ref79] Turner R. J. (2007). Book review. J. Minim. Invasive Gynecol. 14:776. doi: 10.1016/j.jmig.2007.07.001

[ref80] Vaccaro M. Almaatouq A. Malone T. (2024). When combinations of humans and AI are useful: a systematic review and meta-analysis. Nat. Hum. Behav. 8, 2293–2303. doi: 10.1038/s41562-024-02024-1, 39468277 PMC11659167

[ref81] Venkatesh V. Davis F. D. (2000). A theoretical extension of the technology acceptance model: four longitudinal field studies. Manag. Sci. 46, 186–204. doi: 10.1287/mnsc.46.2.186.11926, 19642375

[ref82] Venkatesh V. Morris M. G. Davis G. B. Davis F. D. (2003). User acceptance of information technology: toward a unified view1. MIS Q. 27:540. doi: 10.2307/30036540

[ref83] Venkatesh V. Thong J. Y. L. Xu X. (2012). Consumer acceptance and use of information technology: extending the unified theory of acceptance and use of technology. MIS Q. 36:157. doi: 10.2307/41410412

[ref84] Wang Y. (2008). Assessing e-commerce systems success: a respecification and validation of the DeLone and McLean model of IS success. Inf. Syst. J. 18:68. doi: 10.1111/j.1365-2575.2007.00268.x

[ref85] Wang C. Du Y. Zou B. (2025). Learners’ acceptance and use of multimodal artificial intelligence (AI)-generated content in AI-mediated informal digital learning of English. Int. J. Appl. Linguist. 36:12827. doi: 10.1111/ijal.12827

[ref86] Wijesundara T. R. Xixiang S. (2018). Social networking sites acceptance: the role of personal innovativeness in information technology. Int. J. Bus. Manag. 13:75. doi: 10.5539/ijbm.v13n8p75

[ref87] Wilson A. Burleigh C. (2026). Generative artificial intelligence as a collaborative thought partner: rethinking cognitive partnership, creativity, and knowledge work in the age of intelligent systems. Int. J. AI Pedag. Innov. Learn. Fut. 2026:6963. doi: 10.46787/ijaipil.v2026i1.6963

[ref88] Xia S. (2025) Factors influencing the actual use of generative AI in college English learning: integrating TAM and innovativeness, in: Proceedings of the 2025 6th International Conference on Computer Information and Big Data Applications, CIBDA ‘25. Association for Computing Machinery, New York, pp. 1342–1348.

[ref89] Xiang J. Xing H. (2025). The promotion mechanism of physical and mental health of the elderly in China: the impact of the digital divide and social capital. BMC Public Health 25:2457. doi: 10.1186/s12889-025-23411-x, 40660167 PMC12261863

[ref90] Xu W. Liang H.-N. Yu K. Wen S. Baghaei N. Tu H. (2023). Acceptance of virtual reality exergames among Chinese older adults. Int. J. Hum. Comput. Interact. 39, 1134–1148. doi: 10.1080/10447318.2022.2098559

[ref91] Yan H. Lu L. Li Z. Wang J. (2025) From innovation to implementation: factors driving Chinese farmers’ adoption intentions for laser weeding robots, in: Proceedings of the 2025 International Conference on Smart Agriculture and Artificial Intelligence, SAAI ‘25. Association for Computing Machinery, New York, pp. 150–156.

[ref92] Yang C.-C. Yang S.-Y. Chang Y.-C. (2023). Predicting older adults’ Mobile payment adoption: an extended TAM model. Int. J. Environ. Res. Public Health 20:391. doi: 10.3390/ijerph20021391, 36674145 PMC9859444

[ref93] Yau Y. Shen Y. C. (2025). “Though helpful, still hesitant”: a TAM-based qualitative study on older adults’ ambivalent acceptance and model extensions in AI fitness coaches. Front. Psychol. 16:1666755. doi: 10.3389/fpsyg.2025.1666755, 41479972 PMC12754909

[ref94] Yildirim N. Oh C. Sayar D. Brand K. Challa S. Turri V. . (2023) Creating design resources to scaffold the ideation of AI concepts, in: Proceedings of the 2023 ACM Designing Interactive Systems Conference, DIS ‘23. Association for Computing Machinery, New York, pp. 2326–2346.

[ref95] Yoon S. Kim M. (2023). A study on the improvement direction of artificial intelligence speakers applying DeLone and McLean’s information system success model. Hum. Behav. Emerg. Technol. 2023:458. doi: 10.1155/2023/2683458

[ref96] Zhang C. Shao Y. Yuan Y. Shen W. (2025). Artificial intelligence reshapes creativity: a multidimensional evaluation. Psych. J. 14, 831–840. doi: 10.1002/pchj.70042, 40764253 PMC12702588

[ref97] Zhao L. Fu B. (2024). Assessing the impact of recommendation novelty on older consumers: older does not always mean the avoidance of innovative products. Behav. Sci. 14:473. doi: 10.3390/bs14060473, 38920805 PMC11200579

[ref98] Zhengkun Z. Chuhao G. Shenhui S. Junhao W. Jia W. Wenrui L. (2025). Technology acceptance and service experience of elderly users with AI translation tools. J. Manag. World 2025, 467–472. doi: 10.53935/jomw.v2024i4.715

[ref99] Zhou C. Liu X. Yu C. Tao Y. Shao Y. (2024). Trust in AI-augmented design: applying structural equation modeling to AI-augmented design acceptance. Heliyon 10:e23305. doi: 10.1016/j.heliyon.2023.e23305, 38192792 PMC10771990

[ref100] Zhou Y. Wang Y. Su J. (2025). A study on factors influencing music enthusiast’s behavioral intention to use generative AI for music composition based on an expanded technology acceptance model. Sci. Rep. 15:44802. doi: 10.1038/s41598-025-28532-2, 41462425 PMC12749033

[ref101] Zhu M. Zhao J. Zhu X. Cheng Q. Zhang S. Kong L. (2023). Effects of health-promoting lifestyle on late-onset depression in older adults: mediating effect of meaning in life and Interleukin-6 (IL-6). Psychol. Res. Behav. Manag. 16, 5159–5168. doi: 10.2147/PRBM.S441277, 38146389 PMC10749783

[ref102] Zin K. S. L. T. Kim S. Kim H.-S. Feyissa I. F. (2023). A study on technology acceptance of digital healthcare among older Korean adults using extended TAM (extended technology acceptance model). Admin. Sci. 13:42. doi: 10.3390/admsci13020042

