# Supplementary material for: Psychological and social factors influencing AI painting tool adoption among young-old adults in the context of creative aging
Source: Front Psychol. 2026 May 26;17:1852192. doi: 10.3389/fpsyg.2026.1852192 (PMC13246666; doi:10.3389/fpsyg.2026.1852192)
Supplement: Supplementary file 1 [file Data_Sheet_1.PDF]

Supplementary File

1. Common Method Variance (CMV)

Because all variables were obtained from a single questionnaire, this study first examined the possibility of common method variance (CMV) to reduce potential bias in the statistical analysis (Lindell and Whitney, 2001). The Harman's single-factor test was applied, and all measurement items were entered into an exploratory factor analysis using SPSS 27.0. The results showed that 10 factors were extracted, and the first factor accounted for 29.186% of the total variance, which is below the 40% threshold (see Table S1).

In addition, a single-factor confirmatory factor analysis (CFA) was conducted for further verification. When all items were loaded onto one common factor, the model showed a poor fit (χ^2/df = 8.168, GFI = 0.603, RMSEA = 0.126, RMR=0.275, CFI = 0.510, NFI = 0.480, and NNFI = 0.476), suggesting that the measurement items did not reflect a single common latent factor (see Table S2). Taken together, the data do not appear to suffer from a serious common method variance issue.

Table S1. Common method variance test (Harman's single-factor test)

NO	Initial Eigenvalues (Unrotated)			Extraction Sums of Squared Loadings		
	Total	% of Variance	Cumulative %	Total	% of Variance	Cumulative %
1	9.339	29.186	29.186	9.339	29.186	29.186

Note: Harman's single-factor test is interpreted based on the unrotated initial eigenvalues. A total of 10 factors with eigenvalues greater than 1 were extracted, and the first factor accounted for 29.186% of the total variance, which is below the 40% threshold.

Table S2. Fit indices of the single-factor CFA model for common method variance assessment

**Fit Index**	X^2	*df*	*p*	χ^2/df	**GFI**	**RMSEA**	**RMR**	**CFI**	**NFI**	**NNFI**
**Recommended Threshold**	-	-	>0.05	<3	>0.9	<0.10	<0.05	>0.9	>0.9	>0.9
**Value**	3789.993	464	0.000	8.168	0.603	0.126	0.275	0.510	0.480	0.476
**Other Indices**	**TLI**	**AGFI**	**IFI**	**PGFI**	**PNFI**	**PCFI**	**SRMR**	**RMSEA 90% CI**		
**Recommended Threshold**	>0.9	>0.9	>0.9	>0.5	>0.5	>0.5	<0.1	-		
**Value**	0.476	0.548	0.512	0.530	0.449	0.477	0.103	0.113-0.132		

Note: CFA = confirmatory factor analysis; GFI = goodness-of-fit index; RMSEA = root mean square error of approximation; RMR = root mean square residual; CFI = comparative fit index; NFI = normed fit index; NNFI = non-normed fit index; TLI = Tucker–Lewis index; AGFI = adjusted goodness-of-fit index; IFI = incremental fit index; PGFI = parsimony goodness-of-fit index; PNFI = parsimony normed fit index; PCFI = parsimony comparative fit index; SRMR = standardized root mean square residual; CI = confidence interval.

2. Model Fit Evaluation

To assess the fit of the proposed model, this study employed the standardized root mean square residual (SRMR) and the normed fit index (NFI) as the primary evaluation criteria (see Table S3). Model fit reflects how well the proposed model represents the observed data and is therefore essential for validating the accuracy of the model interpretation. In this study, the SRMR value was 0.040, suggesting a satisfactory level of fit. In general, an SRMR value below 0.080 indicates an acceptable fit, whereas a value lower than 0.050 reflects a particularly good fit between the model and the data (Henseler et al., 2015). These results imply that the model explains the relationships among the variables effectively, with only a small degree of residual error. In addition, the NFI was used as a comparative fit measure, with values closer to 1 indicating better model performance relative to the null model (Akram et al., 2022). In this study, the NFI value of 0.822 was below the conventional 0.90 benchmark (Aghimien et al., 2022) and should therefore be interpreted cautiously. Accordingly, NFI was reported only as supplementary fit information and was not used as the sole evidence of model adequacy. Model evaluation in this study relied primarily on SRMR, measurement model quality, structural path significance, explanatory power, predictive relevance, and PLSpredict results.

Table S3. Model fit.

Fit Index	Computed Values	Threshold Reference
SRMR	0.040	(Henseler et al., 2015)
NFI	0.822	(Akram et al., 2022)

3. Measurement Model Assessment

3.1 Reliability and Validity Test

In PLS-SEM analysis, we focus on indicator reliability, internal consistency reliability, convergent validity, and discriminant validity (Craig et al., 2003). Factor loadings indicate the degree to which the measured variable reflects the information in the latent variable, with larger values (closer to 1) indicating greater representational power. As shown in Table S4, the factor loadings of the measurement variables were all above 0.7, and the factor loadings of the items ranged from 0.828 to 0.886. These values indicate satisfactory indicator reliability and provide initial support for the adequacy of the measurement model.

In this study, internal consistency reliability was evaluated using Cronbach's alpha and composite reliability (CR), while convergent validity was assessed using average variance extracted (AVE). Discriminant validity was examined separately in Section 3.2.

The Cronbach's alpha values for all constructs were higher than 0.80, demonstrating satisfactory internal consistency, with values ranging from 0.810 (IU and PIN) to 0.896

(PU). In addition, composite reliability was examined to provide a more robust assessment of construct reliability. All CR values were above 0.85, further confirming strong reliability, and the CR (rho_c) values ranged from 0.884 (TR) to 0.927 (PU). Convergent validity was then assessed through AVE, and all AVE values exceeded the recommended threshold of 0.50, thus indicating adequate convergent validity. Specifically, the AVE values ranged from 0.718 (TR) to 0.762 (PU). These results suggest that the constructs demonstrated satisfactory internal consistency reliability and adequate convergent validity. Overall, the measurement model showed satisfactory indicator reliability, internal consistency reliability, and convergent validity. Detailed results are presented in Table S4 and Table S5.

Table S4. Reliability and validity test

Construct	Item	Factor Loading	Cronbach's alpha	CR (rho_a)	CR (rho_c)	AVE
HM	HM1	0.853	0.813	0.815	0.889	0.728
	HM2	0.860				
	HM3	0.846				
IS	IS1	0.866	0.836	0.836	0.902	0.753
	IS2	0.861				
	IS3	0.876				
IU	IU1	0.842	0.810	0.814	0.887	0.724
	IU2	0.858				
	IU3	0.854				
OQ	OQ1	0.850	0.820	0.821	0.893	0.736
	OQ2	0.865				
	OQ3	0.859				
PEOU	PEOU1	0.867	0.885	0.886	0.920	0.743
	PEOU2	0.853				
	PEOU3	0.856				
	PEOU4	0.871				
PI	PI1	0.861	0.827	0.827	0.897	0.743
	PI2	0.864				
	PI3	0.861				
PIN	PIN1	0.850	0.810	0.813	0.888	0.725
	PIN2	0.862				
	PIN3	0.842				
PU	PU1	0.865	0.896	0.898	0.927	0.762
	PU2	0.873				

	PU3	0.866				
	PU4	0.886				
RA	RA1	0.854	0.819	0.826	0.892	0.734
	RA2	0.836				
	RA3	0.879				
TR	TR1	0.828	0.806	0.823	0.884	0.718
	TR2	0.878				
	TR3	0.836				

Table S5. Cross-loadings of measurement items.

	**HM**	**IS**	**IU**	**OQ**	**PEOU**	**PI**	**PIN**	**PU**	**RA**	**TR**
HM1	**0.853**	0.221	0.183	0.216	0.266	0.227	0.221	0.283	0.331	0.253
HM2	**0.860**	0.221	0.271	0.222	0.312	0.259	0.249	0.287	0.265	0.216
HM3	**0.846**	0.222	0.239	0.249	0.294	0.24	0.231	0.269	0.275	0.289
IS1	0.196	**0.866**	0.296	0.219	0.409	0.307	0.361	0.237	0.267	0.229
IS2	0.240	**0.861**	0.248	0.216	0.367	0.306	0.283	0.292	0.310	0.210
IS3	0.240	**0.876**	0.263	0.198	0.369	0.308	0.300	0.294	0.293	0.232
IU1	0.204	0.240	**0.842**	0.195	0.410	0.225	0.181	0.396	0.164	0.194
IU2	0.232	0.250	**0.858**	0.245	0.488	0.226	0.256	0.403	0.197	0.182
IU3	0.257	0.300	**0.854**	0.163	0.469	0.241	0.239	0.384	0.198	0.195
OQ1	0.212	0.183	0.202	**0.850**	0.236	0.135	0.227	0.308	0.230	0.298
OQ2	0.228	0.228	0.222	**0.865**	0.217	0.134	0.280	0.338	0.200	0.307
OQ3	0.250	0.215	0.185	**0.859**	0.194	0.136	0.212	0.324	0.231	0.271
PEOU1	0.329	0.363	0.454	0.205	**0.867**	0.340	0.340	0.439	0.309	0.260
PEOU2	0.300	0.377	0.479	0.257	**0.853**	0.338	0.285	0.431	0.303	0.213
PEOU3	0.259	0.352	0.419	0.199	**0.856**	0.342	0.303	0.412	0.310	0.202
PEOU4	0.289	0.421	0.494	0.207	**0.871**	0.392	0.332	0.412	0.289	0.284
PI1	0.244	0.312	0.212	0.154	0.364	**0.861**	0.298	0.242	0.227	0.155
PI2	0.257	0.306	0.228	0.152	0.337	**0.864**	0.293	0.255	0.227	0.153
PI3	0.234	0.295	0.261	0.100	0.359	**0.861**	0.304	0.217	0.273	0.134
PIN1	0.227	0.295	0.254	0.255	0.300	0.297	**0.850**	0.291	0.248	0.206
PIN2	0.268	0.319	0.210	0.228	0.329	0.319	**0.862**	0.314	0.300	0.236
PIN3	0.201	0.312	0.218	0.235	0.303	0.265	**0.842**	0.263	0.230	0.253
PU1	0.248	0.259	0.353	0.321	0.388	0.223	0.246	**0.865**	0.276	0.276
PU2	0.309	0.292	0.413	0.333	0.444	0.258	0.321	**0.873**	0.302	0.308
PU3	0.291	0.267	0.423	0.331	0.444	0.229	0.325	**0.866**	0.319	0.312
PU4	0.293	0.283	0.420	0.330	0.435	0.253	0.292	**0.886**	0.334	0.305
RA1	0.306	0.270	0.158	0.248	0.288	0.214	0.270	0.274	**0.854**	0.310

RA2	0.275	0.306	0.211	0.199	0.297	0.212	0.280	0.281	**0.836**	0.241
RA3	0.291	0.284	0.194	0.214	0.315	0.289	0.241	0.347	**0.879**	0.245
TR1	0.234	0.212	0.190	0.306	0.187	0.125	0.248	0.281	0.253	**0.828**
TR2	0.233	0.230	0.181	0.280	0.277	0.141	0.211	0.336	0.284	**0.878**
TR3	0.291	0.212	0.201	0.287	0.236	0.171	0.240	0.251	0.241	**0.836**

3.2 Discriminant Validity

According to the discriminant validity results shown in Table S6, the square root of the AVE for each latent construct was compared with the corresponding correlation coefficients among constructs (Henseler et al., 2015). The bold values on the diagonal represent the square root of the AVE for each construct, whereas the off-diagonal entries denote the inter-construct correlations. Discriminant validity is considered adequate when the square root of a construct's AVE exceeds its correlations with other constructs. As presented in Table S6, this condition was satisfied for all latent variables. These findings indicate that each construct is empirically distinct from the others and possesses sufficient independent explanatory capability. Therefore, the measurement model demonstrates satisfactory discriminant validity and fulfills the requirements of structural equation modeling, thereby enhancing the credibility of the study results.

Table S6 Discriminant validity analyses (Fornell-Larcker criterion)

	HM	IS	IU	OQ	PEOU	PI	PIN	PU	RA	TR
HM	0.853									
IS	0.260	0.868								
IU	0.272	0.310	0.851							
OQ	0.268	0.243	0.237	0.858						
PEOU	0.342	0.440	0.537	0.252	0.862					
PI	0.284	0.353	0.271	0.157	0.410	0.862				
PIN	0.274	0.363	0.266	0.281	0.366	0.346	0.851			
PU	0.328	0.316	0.463	0.377	0.492	0.276	0.341	0.873		
RA	0.339	0.334	0.220	0.256	0.351	0.281	0.306	0.354	0.857	
TR	0.295	0.258	0.223	0.341	0.279	0.171	0.272	0.345	0.308	0.848

To strengthen the assessment of discriminant validity, this study employed the Heterotrait–Monotrait ratio (HTMT) in addition to the Fornell–Larcker criterion. HTMT is used to examine the relationships among different constructs in relation to the consistency of indicators within the same construct, and values lower than 0.85 or 0.90 are typically regarded as acceptable. The results showed that all HTMT values in this study were below 0.85, indicating satisfactory discriminant validity for all

constructs (see Table S7). Combined with the Fornell–Larcker results, the HTMT findings provide further support for the adequacy of the measurement model, highlight the distinctiveness of the latent variables, and enhance confidence in the robustness of the model estimates.

Table S7. Discriminant validity (HTMT values)

	HM	IS	IU	OQ	PEOU	PI	PIN	PU	RA	TR
HM	-									
IS	0.315	-								
IU	0.332	0.376	-							
OQ	0.328	0.293	0.289	-						
PEOU	0.401	0.51	0.631	0.295	-					
PI	0.346	0.425	0.331	0.191	0.478	-				
PIN	0.335	0.44	0.327	0.344	0.431	0.421	-			
PU	0.382	0.365	0.541	0.439	0.551	0.32	0.397	-		
RA	0.417	0.404	0.268	0.314	0.412	0.338	0.376	0.409	-	
TR	0.369	0.313	0.278	0.421	0.325	0.210	0.340	0.400	0.379	-

Table S8. HTMT Inference (2.5-97.5%)

	HM	IS	IU	OQ	PEOU	PI	PIN	PU	RA	TR
HM	-									
IS	0.212-0.414	-								
IU	0.226-0.435	0.271-0.477	-							
OQ	0.222-0.432	0.183-0.392	0.186-0.391	-						
PEOU	0.306-0.494	0.419-0.598	0.551-0.702	0.196-0.395	-					
PI	0.237-0.451	0.330-0.512	0.227-0.429	0.091-0.296	0.381-0.572	-				
PIN	0.231-0.437	0.344-0.536	0.227-0.429	0.234-0.443	0.337-0.519	0.323-0.515	-			
PU	0.284-0.478	0.271-0.450	0.453-0.623	0.346-0.523	0.471-0.624	0.220-0.414	0.302-0.486	-		
RA	0.319-0.510	0.311-0.492	0.163-0.369	0.213-0.413	0.315-0.505	0.236-0.441	0.271-0.476	0.316-0.498	-	
TR	0.264-0.470	0.207-0.419	0.174-0.381	0.317-0.517	0.220-0.426	0.104-0.316	0.239-0.438	0.307-0.487	0.276-0.474	-

Taken together, these findings provide additional support for the discriminant validity of the measurement model. Although several perception- and experience-related constructs in technology acceptance research may be conceptually adjacent, the present

results suggest that they were adequately differentiated in this study. In particular, the cross-loadings, HTMT values, and HTMT inference results support the empirical distinctiveness of OQ, HM, and PU, which reflect evaluations of generated outputs, intrinsic enjoyment during engagement, and instrumental utility, respectively (see Table S8).
